# Multi-omics approach reveals dysregulated genes during hESCs neuronal differentiation exposure to paracetamol

**DOI:** 10.1016/j.isci.2023.107755

**Published:** 2023-08-28

**Authors:** Mari Spildrejorde, Athina Samara, Ankush Sharma, Magnus Leithaug, Martin Falck, Stefania Modafferi, Arvind Y.M. Sundaram, Ganesh Acharya, Hedvig Nordeng, Ragnhild Eskeland, Kristina Gervin, Robert Lyle

**Affiliations:** 1 PharmaTox Strategic Research Initiative, Faculty of Mathematics and Natural Sciences, University of Oslo, Oslo, Norway; 2 Department of Medical Genetics, Oslo University Hospital and University of Oslo, Oslo, Norway; 3 Institute of Clinical Medicine, Faculty of Medicine, University of Oslo, Oslo, Norway; 4 Division of Clinical Paediatrics, Department of Women’s and Children’s Health, Karolinska Institutet, Stockholm, Sweden; 5 Astrid Lindgren Children′s Hospital, Karolinska University Hospital, Stockholm, Sweden; 6 Department of Informatics, University of Oslo, Oslo, Norway; 7 Department of Molecular Medicine, Institute of Basic Medical Sciences, Faculty of Medicine, University of Oslo, Oslo, Norway; 8 Department of Biosciences, University of Oslo, Oslo, Norway; 9 Division of Obstetrics and Gynecology, Department of Clinical Science, Intervention and Technology (CLINTEC), Karolinska Institutet, Alfred Nobels Allé 8, SE-14152 Stockholm, Sweden; 10 Center for Fetal Medicine, Karolinska University Hospital, SE-14186 Stockholm, Sweden; 11 Pharmacoepidemiology and Drug Safety Research Group, Department of Pharmacy, University of Oslo, Oslo, Norway; 12 Division of Clinical Neuroscience, Department of Research and Innovation, Oslo University Hospital, Oslo, Norway; 13 Centre for Fertility and Health, Norwegian Institute of Public Health, Oslo, Norway

**Keywords:** Developmental neuroscience, Neurotoxicology, In vitro toxicology, Stem cells research, Genomics, Transcriptomics

## Abstract

Prenatal paracetamol exposure has been associated with neurodevelopmental outcomes in childhood. Pharmacoepigenetic studies show differences in cord blood DNA methylation between unexposed and paracetamol-exposed neonates, however, causality and impact of long-term prenatal paracetamol exposure on brain development remain unclear. Using a multi-omics approach, we investigated the effects of paracetamol on an *in vitro* model of early human neurodevelopment. We exposed human embryonic stem cells undergoing neuronal differentiation with paracetamol concentrations corresponding to maternal therapeutic doses. Single-cell RNA-seq and ATAC-seq integration identified paracetamol-induced chromatin opening changes linked to gene expression. Differentially methylated and/or expressed genes were involved in neurotransmission and cell fate determination trajectories. Some genes involved in neuronal injury and development-specific pathways, such as *KCNE3*, overlapped with differentially methylated genes previously identified in cord blood associated with prenatal paracetamol exposure. Our data suggest that paracetamol may play a causal role in impaired neurodevelopment.

## Highlights

• Multi-omics analyses of paracetamol’s effects on hESC neuronal differentiation
• Single-cell data show dose-dependent changes in cell proliferation and maturation
• Some overlap with differentially methylated genes of prenatal exposed cord blood
• Visualize and analyze single-cell gene expression and chromatin states in webtools

## Graphical abstract

**Figure:**
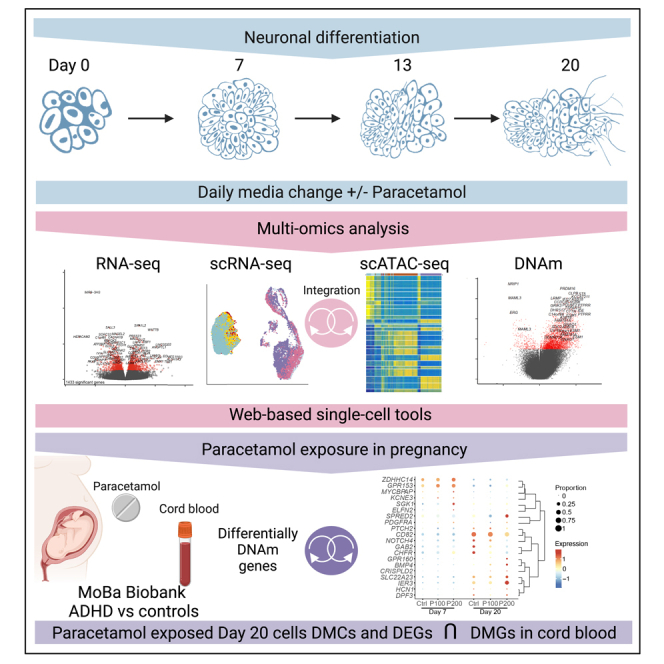

## Introduction

Paracetamol (also known as acetaminophen) is the most widely used analgesic and antipyretic during pregnancy, and it is considered safe for use as the first line option for pregnant women in need of mild analgesics or antipyretics.^1^^,^^2^^,^^3^^,^^4^ A number of large epidemiological studies have reported an association between long-term maternal paracetamol use during pregnancy and increased risk of adverse neurodevelopmental outcomes, such as attention deficit/hyperactivity disorder (ADHD), in the child.^5^^,^^6^^,^^7^^,^^8^^,^^9^^,^^10^^,^^11^^,^^12^^,^^13^ The association is reported to be stronger with long-term exposure and higher dose.^14^ In 2019, the EUs pharmacovigilance safety committee (PRAC) reviewed all the available evidence, including non-clinical and epidemiological studies, regarding the impact of prenatal paracetamol exposure on impaired neurodevelopment in offspring. PRAC concluded that the available evidence is inconclusive, and recommended that the summary of product characteristics (SmPC) of paracetamol containing products should be updated to reflect the current state of scientific knowledge; “*Epidemiological studies on neurodevelopment in children exposed to paracetamol in utero show inconclusive results*” (PRAC, 2019).^15^ More recently, a group of researchers published a consensus statement and literature review in Nature Reviews Endocrinology concluding that there is growing evidence supporting the hypothesis that *in utero* exposure to paracetamol can impair fetal development.^16^ This conclusion is still highly debated and contested by others (ENTIS, 2021)^17^^,^^18^^,^^19^ reflecting a need for further research.

Using samples from the Norwegian Mother, Father and Child cohort (MoBa) biobank, we have previously shown differences in DNA methylation (DNAm) in cord blood from children diagnosed with ADHD who have been exposed to paracetamol (>20 days) during prenatal development compared to unexposed children.^20^ These findings suggest that DNAm might be involved in the pathogenesis of ADHD, but the causality and effect on neuronal differentiation and brain development is not known. It is well established that normal prenatal neurodevelopment involves cellular differentiation and establishment of cell-type specific epigenetic patterns, and that these events are prone to influences by environmental factors. For instance, maternal smoking has been shown to induce DNAm changes and modulate risk of neurodevelopmental disorders (NDDs).^21^^,^^22^^,^^23^ If and how paracetamol modulates the apparent increased risk of NDDs is currently unknown.

Although paracetamol has been used for more than a hundred years, the mechanisms of action still remain unclear and appear to involve numerous physiological pathways, that vary among *in vitro* and *in vivo* studies.^24^^,^^25^ Following administration of therapeutic doses, paracetamol is primarily metabolized into pharmacologically inactive glucuronide and sulfate conjugates, while a small portion is oxidized to form the highly reactive metabolite, NAPQI.^26^ Although the precise neurotoxic mechanism of action for paracetamol and its metabolites remains unclear, it is known that deacetylation of paracetamol by N-deacetylase yields *p*-aminophenol, later conjugated with arachidonic acid by FAAH to form the active metabolite N-arachidonoylphenolamine (AM404).^27^ Paracetamol, once it crosses the blood-brain barrier,^28^ may provide neuroprotection at low doses, whereas high doses may induce neurotoxicity,^29^ possibly through oxidative stress. Furthermore, to our knowledge no studies have addressed whether and how the different cell populations derived from hESCs during early neuronal differentiation could potentially metabolize paracetamol and form bioactive compounds.

Recently, we established a protocol for neuronal differentiation of human embryonic stem cells (hESCs), which can be used in neuropharmacological studies.^30^ In the present study, we have used this model system of early human brain development and investigated the effects of paracetamol exposures on transcriptional and epigenetic regulation. Paracetamol doses were selected to reflect therapeutic maternal doses and fetal *in utero* exposures.^31^^,^^32^^,^^33^ By integrating multiple omics methods (bulk RNA-seq, bulk DNAm, single-cell RNA-seq and ATAC-seq, Table 1) we observed time and dose effects of paracetamol exposure during neuronal differentiation.

**Table 1:** Overview over presented datasets

Day	Control	P100	P200
0	RNA-seq (5), DNAm (6)		
7	RNA-seq (6), DNAm (6), scRNA-seq (2)	RNA-seq (5), DNAm (6), scRNA-seq (2)	RNA-seq (5), DNAm (5), scRNA-seq (2)
13	RNA-seq (4), DNAm (4)		
20	RNA-seq (4), DNAm (6), scRNA-seq (2), scATAC-seq (1)	RNA-seq (4), DNAm (6), scRNA-seq (2), scATAC-seq (1)	RNA-seq (6), DNAm (4), scRNA-seq (2), scATAC-seq (1)

P100; 100 μM paracetamol, P200; 200 μM paracetamol. Number of replicates are shown in parentheses.

## Results

### Neuronal differentiation exposure, timeline, and morphology

**Figure 1:**
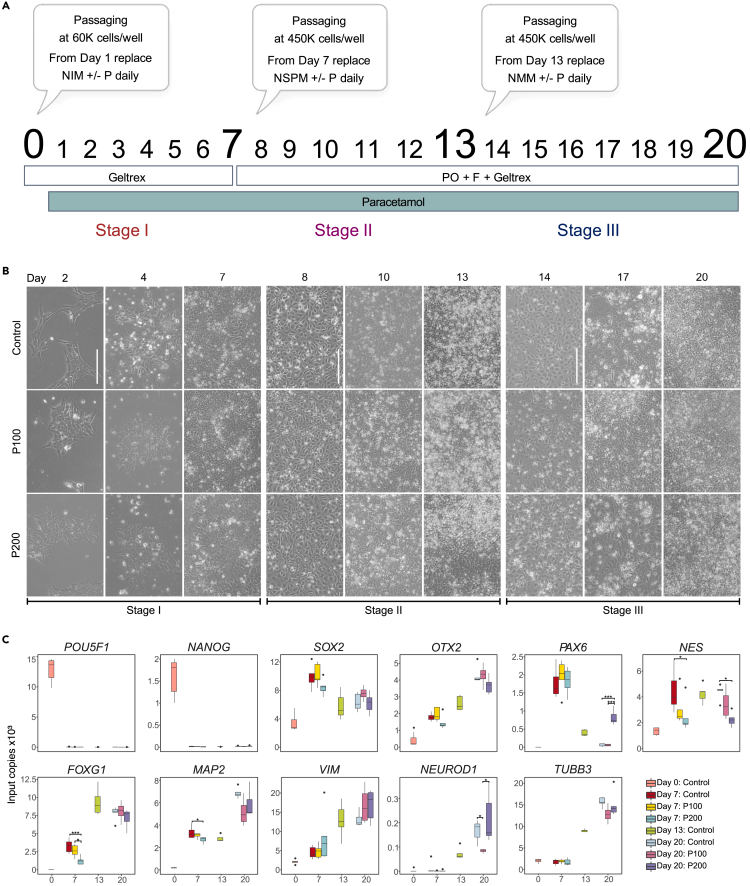
Neuronal differentiation of hESC to model neurodevelopmental effects of paracetamol (A) Schematic illustration of the different phases of the neuronal differentiation protocol from Day 0 to Day 20. Cells were exposed to 100 or 200 μM paracetamol from Day 1 and onwards. The effect of paracetamol on gene expression and epigenetic profiles was evaluated at Day 7 and Day 20. (B) Representative brightfield images of the differentiation timeline for control, P100 or P200 cells during differentiation at Day 2, 4, 7, 8, 10, 13, 14, 17 and 20 (scale bar corresponds to 100 μM). (C) ddPCR results from 4 to 6 replicates of mRNA expression of selected marker genes from Days 0, 7, 13 and 20. Significant comparisons are marked with asterisks (Student’s *t* test, ∗: p ≤ 0.05, ∗∗∗: p ≤ 0.001).

We investigated epigenetic and transcriptomic effects of exposure to paracetamol using an *in vitro* neuronal differentiation protocol that drives hESCs toward anterior neuroectoderm.^30^^,^^34^ The neuronal differentiation is divided into three stages: the neural induction phase (Stage I) ends at Day 7, the self-patterning phase (Stage II) ends at Day 13, and the FGF2/EGF2-induced maturation phase (Stage III) ends at Day 20 (Figure 1A). We replaced culture media daily, and the cells were exposed to 100 or 200 μM paracetamol during differentiation from Day 1 and onwards. These concentrations have been documented to be in the range of therapeutic plasma concentrations.^31^^,^^32^^,^^33^ Unexposed (control) and paracetamol-exposed cells were harvested for downstream analyses on Day 7 and 20. We also harvested control cells at the onset of differentiation (hESCs; Day 0), and on the intermediate timepoint that cells were passaged (Day 13) to assess whether paracetamol exposed cells had mRNA abundance changes related to proliferation or delayed differentiation.

The timeline of brightfield images of the control cells versus cells exposed to 100 (P100) or 200 μM paracetamol (P200) documents the morphological changes and cell culture density at differentiation Days 2, 4, 7, 8, 10, 13, 14, 17 and 20 (Figure 1B). Tightly packed neuroepithelial cells form the neural rosettes by Day 7 reassembled at the next stage under high density cell passaging and proceeded to maturation. We did not observe any distinct morphological changes in the differentiating cultures exposed to 100 (P100) or 200 μM paracetamol (P200). However, preliminary paracetamol titration experiments showed that exposing the differentiating cells to 400 μM paracetamol increased cell death and unpatterned morphology in cells and were thus discontinued. A set of representative images following the 20-day timeline in control cultures and 100, 200 and 400 μM paracetamol-exposed cells is presented in supplemental information (Figures S1A–S1C).

### Validation of differentiation markers

The effect of paracetamol on gene expression at Days 7 and 20 was assayed using digital droplet PCR (ddPCR) (Figure 1C). Expression of the pluripotency transcription factors (TFs) *POU5F1* and *NANOG* decreased significantly after neural induction. As anticipated, we observed an increase in expression of the neural markers *SOX2*, *OTX2*, *FOXG1*, and *MAP2* on Day 7 and *VIM*, *TUBB3*, and *NEUROD1* on Day 13. Notably, the expression of *FOXG1*, *MAP2*, and *NES* was significantly different between exposed and control cells on Day 7. Differential expression upon paracetamol exposure was also documented for filament *NES*, *PAX6*, and *NEUROD1* on Day 20.

### Paracetamol-induced gene expression changes in genes involved in neural development

**Figure 2:**
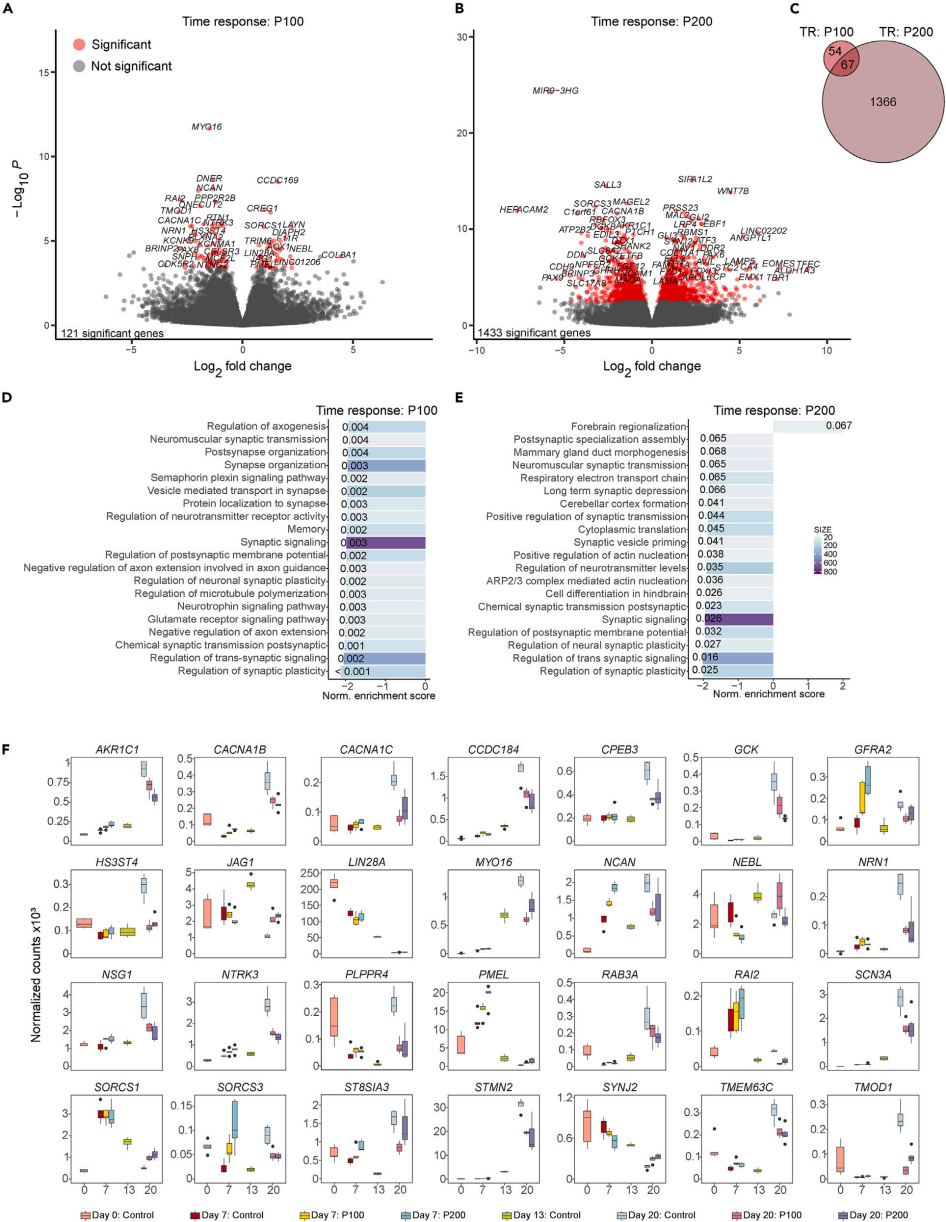
Paracetamol exposure of differentiating hESCs modulates expression of genes involved in neural development (A and B) Volcano plots of longitudinal differences in gene expression difference between Day 7 and Day 20 (Day 20 minus Day 7) in A) P100 and B) P200 cells compared to control cells. Marked in red are significantly differentially expressed genes with an FDR <0.05. (C) Corresponding Venn diagram shows the number of overlapping DEGs between the P100 and P200 time-response (TR) comparisons (Day 20 minus Day 7 compared to control). (D and E) Top 20 enriched BPs based on GSEA in (D) P100 cells and (E) P200 cells compared to control cells over time (Day 20 minus Day 7). Normalized enrichment scores are represented as bars with FDR q-values. (F) Gene expression levels of selected DEGs in both P100 and P200 cells compared to control cells over time (TR) (overlapping area in C).

To delineate the effect on gene expression, we performed bulk gene expression analysis using RNA-seq in controls and paracetamol exposed cells (P100 and P200; Figures 2 and S2; Table S1). Principal component analysis showed that samples clustered according to differentiation day (Figure S2A; STAR Methods). Overall, when we compared gene expression from P100 versus control we identified 121 differentially expressed genes (DEGs, Figure 2A) and 1 433 DEGs between Day 7 and Day 20 P200 versus control (Figure 2B) (Table S1). Of these, 67 DEGs overlapped between P100 and P200 compared to control (Figure 2C). Pairwise comparisons between paracetamol (P100 and P200) and control at each day are shown in Figures S2B and S2C. The bulk RNA-seq analysis of the previously selected marker genes (Figure 1B) correlated well with the ddPCR results of selected marker genes (Figure S1D).

Gene set enrichment analyses (GSEA) identified downregulated biological processes (BPs) involved in synaptic organization, transmission, and regulation in the P100 time-response analysis (Figure 2D). Notably, one upregulated BP in the P200 time-response analysis were enriched for *forebrain regionalization*, whereas downregulated BPs were enriched for *synaptic signaling*, *regulation of* transsynaptic *signaling*, *regulation of synaptic plasticity* and *regulation of neurotransmitter levels* (Figure 2E). Moreover, the DEGs associated with P100 and P200 reflected BP enrichment of transmitter transport and regulation, synaptogenesis, synaptic organization, and plasticity. We also performed g:profiler^35^ analysis of BPs on up and downregulated DEGs for P100 time-response and P200 time-response (Tables S1H and S1I). Top P100 time-response downregulated GO terms were generation of neurons, nervous system development, neuron differentiation and for P200 time-response downregulated GO terms were *nervous system development,* transsynaptic *signaling* and *synaptic signaling* (Tables S1J and S1K). Of the 46 BPs for downregulated DEGs P100 time-response, 40 overlapped with downregulated P200 time-response correlating with many overlapping GO terms identified above. There were no upregulated BPs for P100 time-response DEGs, and top identified BPs for upregulated P200 time-response GO terms were *anatomical structure morphogenesis, animal organ morphogenesis* and *animal organ development* (Table S1J and S1K).

The bulk RNA-seq pairwise comparisons of the P100 and P200 paracetamol-exposed cells to controls, showed an overlap of several DEGs (Figures S2D–S2F). Exposure to paracetamol was associated with downregulation of genes previously linked to migration and neural development (e.g., *NTRK3*, *PMEL*,^36^ *CCDC184*,^37^ *MYT1*^38^^,^^39^) (Figures 2F and S2G). Furthermore, we identified DEGs linked to synaptic organization and transduction (e.g., *MYO16*,^40^ *HS3ST4*^41^*, SORCS3*^42^), metabolism (*GCK*^43^), neuronal survival, dendrite branching, axonal growth and neural projection in development (e.g., *NSG1*,^44^ *TMEM3C6*,^45^ *TMOD1*^46^*, PLPPR4*^47^*, NEBL*,^48^ *NRN1*,^49^ and *GFRA2*^50^) and channelopathies (e.g., *CACNA1B/C*,^51^ *SCN3A*^52^) (Figures 2F and S2C). Thus, these bulk RNA-seq analyses revealed transcriptional dysregulation of genes related to possible developmental delays between control and paracetamol-exposed cells.

### Single-cell RNA sequencing reveals dose-specific changes in several major cellular processes after paracetamol exposure

**Figure 3:**
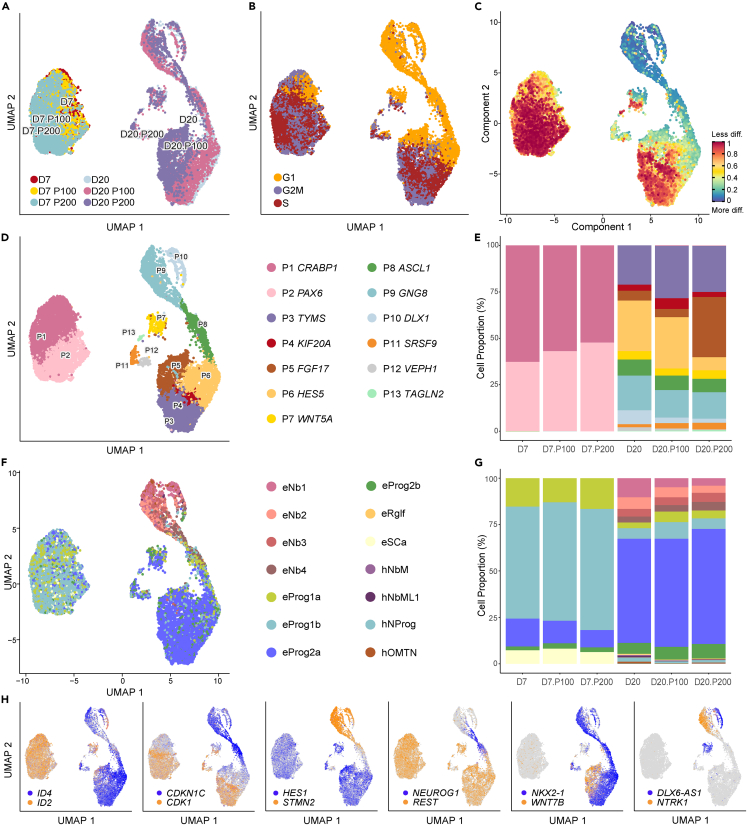
scRNA-seq analysis revealed shifts in cell type composition of differentiating cells exposed to paracetamol (A–E) Day 7 and Day 20 control cells and cells exposed to 100 μM (P100) or 200 μM (P200) paracetamol were visualized with UMAP and colored by (A) sample identity, (B) Seurat predicted cell cycle phase, (C) CytoTRACE pseudotime differentiation trajectory, (D) defined Seurat clusters at resolution 0.4 with corresponding gene annotations and (E) cell proportions per cluster. (F) UMAP plots of cells colored by SingleR cell annotation to Early Human Brain reference with corresponding cell annotations. Cell types starting with “e” are hESC derived cells and cell types starting with “h” are *in vivo* human embryo cell types. Nb1-4; neuroblasts, Prog1-2; neuronal progenitors, Rglf; radial glia-like cells, SCa; stem cells, NbM; medial neuroblasts, NbML1; mediolateral neuroblasts, NProg; neuronal progenitors, OMTN; oculomotor and trochlear nucleus. (G) Corresponding cell proportions per cell type. (H) UMAP plots of dual gene co-expression for *ID2/ID4*, *CDK1/CDKN1C*, *HES1/STMN2, REST/NEUROD1, NKX2.1/WNT7B* and *NTRK.1/DLX6-AS1* indicate that maturation signatures agree with the CytoTRACE trajectory analysis.

To explore cell-type specific gene expression and maturation signatures over time, we performed single-cell RNA sequencing (scRNA-seq) analysis of control and paracetamol-exposed cells (P100 and P200) at Days 7 and 20 (Table S2). Specifically, our aim was to determine whether paracetamol exposure caused deviations in neuronal differentiation compared to control cells. A total of 15 201 cells (n = 6 924 Day 7 cells, n = 8 277 Day 20 cells) from two time-course experiments were aggregated and projected in Uniform Manifold Approximation and Projections (UMAPs, Figure 3; STAR Methods). The scRNA-seq data may also be visualized in the open access webtool (**hescneuroparacet**), where expression of genes can be explored per cell, cluster, and time point.

The cells clustered according to differentiation day and not exposure to paracetamol (Figure 3A). Consistent with previous results,^34^ Seurat-predicted cell cycle phase showed an increase in G1 cells at Day 20 (Figure 3B). However, the existing cell cycle analysis tools are unable to decipher the proportion of neurons that are in G0 phase. Using CytoTRACE pseudotime differentiation trajectory analysis, the most differentiated cells were found at Day 20 (Figure 3C). The composite neuronal differentiation P1-P13 clusters were manually annotated with genes *CRABP1*, *PAX6, TYMS, KIF20A, FGF17, HES5, WNT5A, ASCL1, GNG8, DLX1, SRSF9, VEPH1* and *TAGLN2*, respectively (Figure 3D). At Day 7, we observed a shift in cell composition from P1 to P2 in the cells exposed to paracetamol (Figure 3E). The effect was more prominent at Day 20, albeit not statistically significant (Tables S2I and S2J and STAR Methods),^53^ with a higher proportion of paracetamol-exposed cells compared to controls annotated to cluster P5 and a more prominent effect at P200 exposure, whereas a lower proportion were annotated to P6, P9 and P10 for both concentrations (Figure 3E).

To further investigate cell identity, the data were juxtaposed with a scRNA-seq Human Brain dataset^54^ (STAR Methods and **hescneuroparacet**). Most cells at Day 7 resembled neuronal progenitors, whereas cells at Day 20 were similar to neuronal progenitors, neuroblasts and neurons (Figure 3F). At Day 20, there was a subtle shift from cells annotated as neuroblasts toward the less differentiated neuronal progenitors in paracetamol-exposed cells compared to controls (Figure 3G). UMAP plots of the dual co-expression of the genes *ID2/ID4*, *CDK1/CDKN1C*, *HES1/STMN2, REST/NEUROD1, NKX2-1/WNT7B*, and *NTRK1/DLX6-AS1* indicate agreement between these maturation signatures and the CytoTRACE trajectory analysis (Figures 3C and 3H).

The analysis of the top DEGs per cluster (Figure S4) and data exploration (Figure S5), showed that paracetamol exposure induced changes in several major processes at the selected timepoints. We identified dose-dependent changes that link paracetamol exposure to cell-cycle transition important in neuronal maturation (*MKI67, PCNA, TP53*, *CDK1, MYBL2* and *GJA1)* neural induction differentiation, or its inhibition (*REST, RAX, PAX6, HES1, HES5, ID3* and *ID4),* neurite outgrowth and cortical neurogenesis (*GBA2, ASPM*), neuronal maturation *(CDKN1C, POU2F1, POU3F1, ROBO1, STMN2* and *STMN4)* and WNT and FGF signaling (*FRZB, WNT4, WNT7B, FGF8* and *FGFRL1)* (Figure S5). We observed a differential and dose dependent expression of crucial spatiotemporally regulated transcription factors associated with brain development (e.g., *NKX2.1, OTX2, FOXG1, ASCL1, ISL1, EMX2* and *HOXA1*), neurotransmitter transporter expression (*SAT1*, *SLC2A1*)*.* Finally, we found differential expression of genes related to cellular response to toxic insults (e.g., *DDR1*; Figure S5).

### Paracetamol exposure is associated with differential expression of neural lineage markers

Performing GO analyses, we identified enrichment of BPs after paracetamol exposure in the scRNA-seq datasets. First, we identified the 10 most upregulated and downregulated BPs between control cells and P100 or control cells and P200 cells at Day 7 (Figures S3A and S3B, respectively). Notably, BPs involved in *DNA replication* and *cell-cycle regulation* were upregulated in cells exposed to both paracetamol doses, whereas we identified a downregulation of a BP which involves *generation of neurons*. DEGs in P100 cells compared to control cells were enriched for more neuronal specific annotations. Furthermore, P200 compared to control cells identified upregulated GO terms involved in *cellular responses to toxic insults* and *DNA checkpoint activation*. At Day 7, the relative gene expression of top 20 DEGs between P100 (Figure S3C) and P200 (Figure S3D) is shown. Furthermore, the gene expression of the top overlapping DEGs between the P100 and P200 cells compared to control cells at Day 7 (Figure S3E) identified gene specific similarities. We found changes in crucial genes, such as the master gene of forebrain development *FOXG1*^55^ and genes of the *HES* and *ID* gene families involved in differentiation and neurogenesis.^56^

Next, we extracted the top 10 upregulated and downregulated BPs among the DEGs at Day 20 between control and P100 cells (Figure S3F) or control and P200 cells (Figure S3G). In both comparisons, downregulated GO terms included *neuron/nervous system development* and *microtubule polymerization or depolymerization*. Of the top 20 DEGs at Day 20 between paracetamol-exposed and control cells, we found major patterning TFs, such as *NKX2-1* and *EMX2* (Figures S3H and S3I). Moreover, genes that belong to the ZIC family, among other genes involved in Notch and Wnt signaling, were also identified (Figures S3H and S3I). The gene expression of the top overlapping genes between P100 and P200 cells were compared to control cells at Day 20 (Figure S3J). The analysis of GO terms delineated gene specific changes, such as upregulation of *SELENOW* (Figure S3D)*,* previously associated with neuroprotection from oxidative stress.^57^ Paracetamol exposure induced differentiation lag as evidenced by the *PAX6* expression in some P200 cells (Figure 1C). We also observed a downregulation of tissue- and stage-specific genes, such as *FABP7*, *ISL1, STMN2* and *INA*. In addition, *TUBB1A*, the isotype associated with postmitotic neurons,^58^ and *TUBB2B*, that constitutes 30% of all brain beta tubulins,^59^ were also found among the downregulated genes. Interestingly, *PEG10* and *C1orf61* and *MIR9-1HG* appear in the Day 20 P200 comparisons, genes which have recently been linked to cortical migration and intercellular communication,^60^ further documenting how the P200 dose of paracetamol could affect proper network formation and cell-to-cell signaling.

### Integration of scATAC- and scRNA-seq link paracetamol-induced changes in chromatin opening to transcriptional activity at Day 20

**Figure 4:**
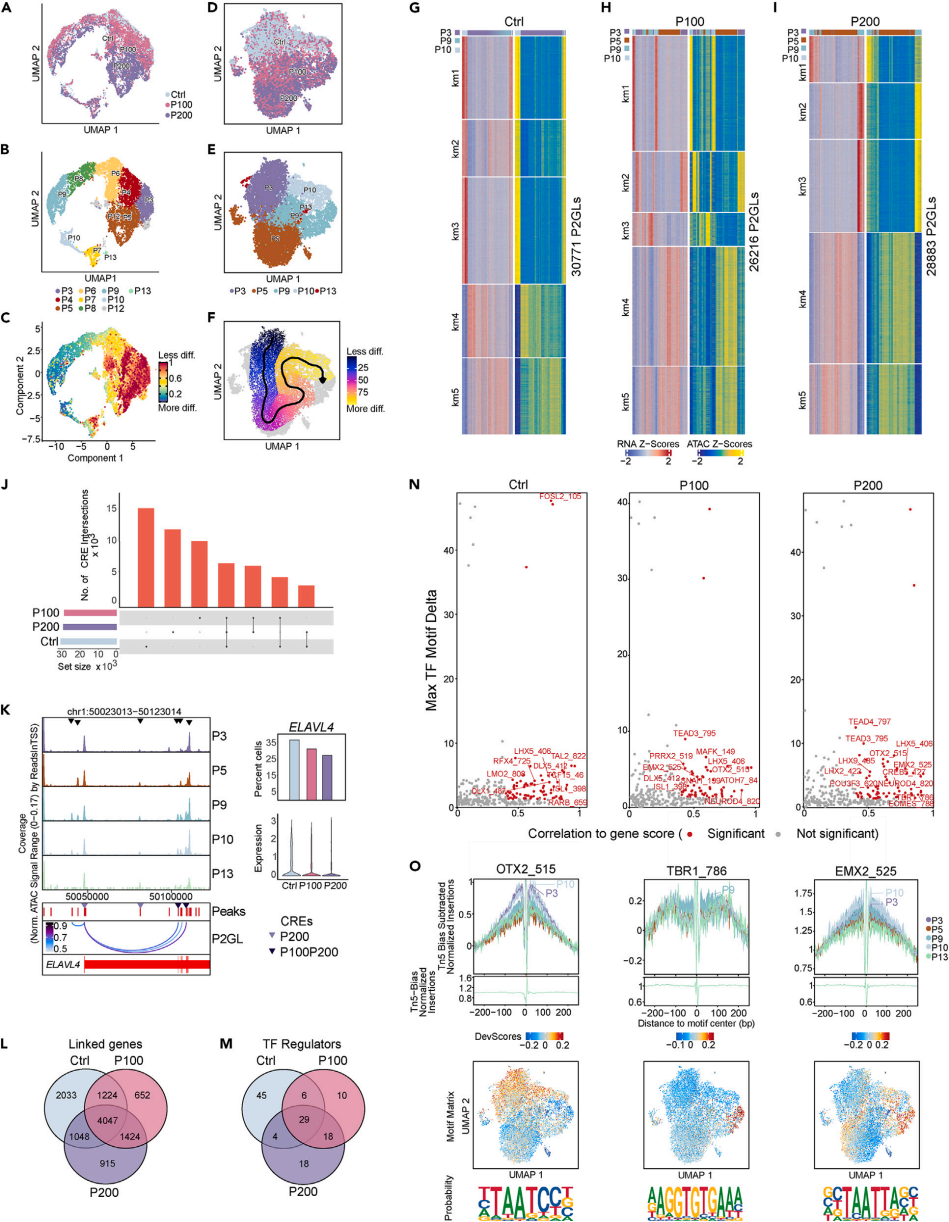
Effects of paracetamol on chromatin accessibility and integration with scRNA-seq at Day 20 (A–C) scRNA-seq UMAP plots colored by A) sample identity, B) scRNA-seq clusters or C) CytoTRACE pseudotime differentiation trajectory. (D and E) scATAC-seq UMAP plots of colored by D) sample identity and E) remapped clusters following constrained alignment of cell populations by scATAC-seq and scRNA-seq integration. (F) Supervised pseudotime trajectory of integrated clusters. (G–I) Heatmaps of scATAC-seq and scRNA-seq side-by-side representing peak to gene links (P2GLs) in G) control cells, H) P100 cells and I) P200 cells. Rows were clustered using k-means (k = 5). (J) Overlap of putative CREs from P2GLs analyses of Day 20 control versus P100 and P200. (K) *ELAVL4* locus browser view (GRCh38.p13) from 5000 cells with ATAC-seq signals in integrated clusters. Differential chromatin opening (black triangles), ATAC peaks (red), P2GLs (arcs) and putative CREs enriched in P200 or P100 and P200 cells (colored triangles) are shown. The bar plot to the right shows the corresponding percentage of cells expressing *ELAVL4*, and the plot below represent the expression levels. (L) A Venn diagram representing overlap of linked genes. (M) Venn diagram of TF regulators identified from P2GLs integrative analysis. (N) A selection of positive TF regulators computed using gene integration scores with motifs in the putative CREs. (O) Footprints for selected TF regulators OTX2, TBR1 and EMX2 demonstrating preferential opening per cluster; below are the corresponding ArchR motif deviation scores, motif matrix and representative sequence logos identified in the scATAC-seq dataset.

To understand whether paracetamol exposure during differentiation influenced the chromatin landscapes at Day 20 we performed single-cell assay for transposase-accessible chromatin using sequencing (scATAC-seq).^61^ We obtained 3 042 nuclei for Day 20 control and 3 480 and 4 282 nuclei for Day 20 exposed to 100 μM (P100) and 200 μM paracetamol (P200), respectively. First, we reanalysed scRNA-seq data from Day 20 controls, P100 and P200, and remapped the P3-P13 clusters (Figures 4A and 4B and webtool **hescneurodiffparacet**). The maturation trajectory cohered with the initial Day 7-Day 20 time point analysis (Figures 3C and 4C). Next, we mapped the scATAC-seq data (Figure 4D) to 15 scATAC-seq clusters (C1-C15; Figure S6A) that we integrated with the annotated scRNA-seq P3, P5, P9, P10 and P13 clusters (Figure 4E). The quality of the combined scATAC-seq datasets was documented with an even distribution of integrated clusters (P3, P5, P9 and P10) over TSS, promoters, exons, introns, and distal genomic regions (Figures S6B and S6C). The P13 cluster is represented by very few nuclei and displayed lower enrichment across all genomic regions.

To further explore the differential chromatin accessibility across the genomes, we correlated distal accessible regions to gene expression. *Cis*-regulatory interactions with active genes were predicted by the integrative single-cell analysis using ArchR.^62^ This allows for identification of putative cis regulatory element (CREs) – gene pairs (peak-to-gene links (P2GLs) for biological and functional comparisons of Day 20 control cells with P100 and P200 exposed cells. We grouped P2GLs into five clusters and plotted heatmaps of gene expression and gene scores for all three datasets (P2GLs represent actively expressed genes linked to chromatin opening, Figures 4G–4I). Heatmaps show differential gene expression to the left, and gene scores for chromatin opening to the right, for CRE-gene pairs across cell clusters P3, P9 and P10 for control, and P3, P5, P9 and P10 for P100 and P200. The rows represent k-means 1–5 and are based on z-score-scaled associated gene expression levels. The scores for scRNA-seq and scATAC-seq were nicely correlating within the k-mean clusters showing good integration of the two modalities. When we integrated the scATAC-seq and the scRNA-seq datasets for Day 20, the clusters P3, P5, P9 and P10 and P13 were mapped (Figure 4E). Notably, the heatmaps of control and P100 and P200 cells were different with an absence or presence of the integrated cell cluster P5, also described as having differential prominence in the scRNA-seq data (Figure 3E).

We observed relatively similar numbers of putative CREs in controls (n = 30,771), P100 (n = 26,216) and P200 (n = 28,883) (Figures 4G–4I). Only 6,960 of the putative CREs overlapped between the three datasets, whereas 4,732, 3,435, and 6,534 putative CREs specifically overlapped between control and P100, control and P200, and P100 and P200, respectively (Figure 4J; Tables S3A–S3C). Moreover, we observed that many of the putative CREs were only detected in individual datasets (Day 20 control; n = 15 644, P100; n = 10 460 and P200; n = 12 304 CREs). These variations in putative CREs suggests that paracetamol exposure results in changes in chromatin accessibility. For example, in the locus of the neuronal marker gene *STMN2,* which showed higher gene expression in unexposed cells compared to exposed cells, showed changes in chromatin opening peaks in paracetamol exposed cells (Figures S3J, S5, and S6D). Furthermore, accessibility peaks in the *ELAVL4* locus displayed differential chromatin opening in clusters P3, P5, P9, and P10. We also identified putative CREs in the exposed cells that correlated with higher *ELAVL4* expression in control cells compared to P100 or P200 cells (Figure 4K; Tables S3A–S3C).

### Paracetamol affects region-specific chromatin accessibility

The putative enhancer-gene interactions identified likely represent chromatin regulomes in the control and the paracetamol-exposed cells. We therefore compared the level of overlap of linked genes to better understand the effect of paracetamol exposure on chromatin regulation. Linked genes are defined as actively expressed genes by scRNA-seq that have linked chromatin accessibility regions in proximity to the gene locus. A larger proportion of linked genes overlapped between the Day 20 control and paracetamol exposed cells (n = 4,047), and the P100 (n = 1 224) and P200 (n = 1,048) cells (Figure 4L; Tables S3A–S3C and S3V). GO analyses of the linked genes revealed a common enrichment of BPs, such as *nervous system development*, *biological regulation, neurogenesis,* and *neuron differentiation*, varying in the different k-means (km) clusters (Figure S6D; Tables S3D–S3F). Linked genes identified in paracetamol exposed cells (P100, n = 652; P200, n = 915 and 1424 for both P100 and P200) indicate exposure-induced changes in gene expression. Interestingly, linked genes including neuronal lineage transcription factors (e.g., *PAX6*, *NEUROD4, NEUROG1*, *SOX9*, and *SOX2*) and genes involved in chromatin modification (e.g., histone H3K27 acetyltransferase *EP300*,^63^ Histone H3K27me3 demethylase *KDM6A*^64^ and chromatin remodeller SNF2 subfamily member *SMARCAD1*^65^), suggest that paracetamol may contribute to modulation of transcriptional regulation and chromatin structure (Table S3V). In addition, GO analysis of the putative CREs identified enrichment of BPs per k-means group and sample are shown in Tables S3G–S3U.

Enriched TF motifs in the CREs may infer binding events that regulate gene expression programs. We identified TFs (n = 29) that were common for control, P100 and P200 cells, including NEUROD1, NEUROG1, SOX9, HMGA1 and members of the ONECUT and NHLH families, which have previously been described in the neuronal differentiation protocol.^34^ TFs were found to be common between P100 and P200 cells (n = 18) or dependent on paracetamol dose (Figures 4M, 4N, S3I, and S6F; Table S4). The TF footprinting predicts binding events due to the protection of the TF from Tn5 transposition in accessible chromatin.^62^ Based on the differential TF enrichment in P100 and P200 cells (Figure 4N), we generated TF footprints for OTX2, TBR1, and EMX2 as aggregate of genome-wide binding sequences adjusted for Tn 5 bias in the different integrated clusters (Figure 4O). Moreover, the UMAP plots for Motif Matrix show that these factors have binding events that are enriched in open chromatin in different integrated clusters. The OTX2 motifs were more enriched in clusters P3 and P10*,* whereas TBR1 motifs were mainly detected in cluster P9 and EMX2 motifs were found in clusters P9 and P10 (Figure 4O) suggesting that these TFs may potentially have cluster specific gene expression regulation. The computed sequence logos identified for these factors across enriched clusters are shown below each TF (Figure 4O).

### Paracetamol induced changes in DNAm during neuronal differentiation

**Figure 5:**
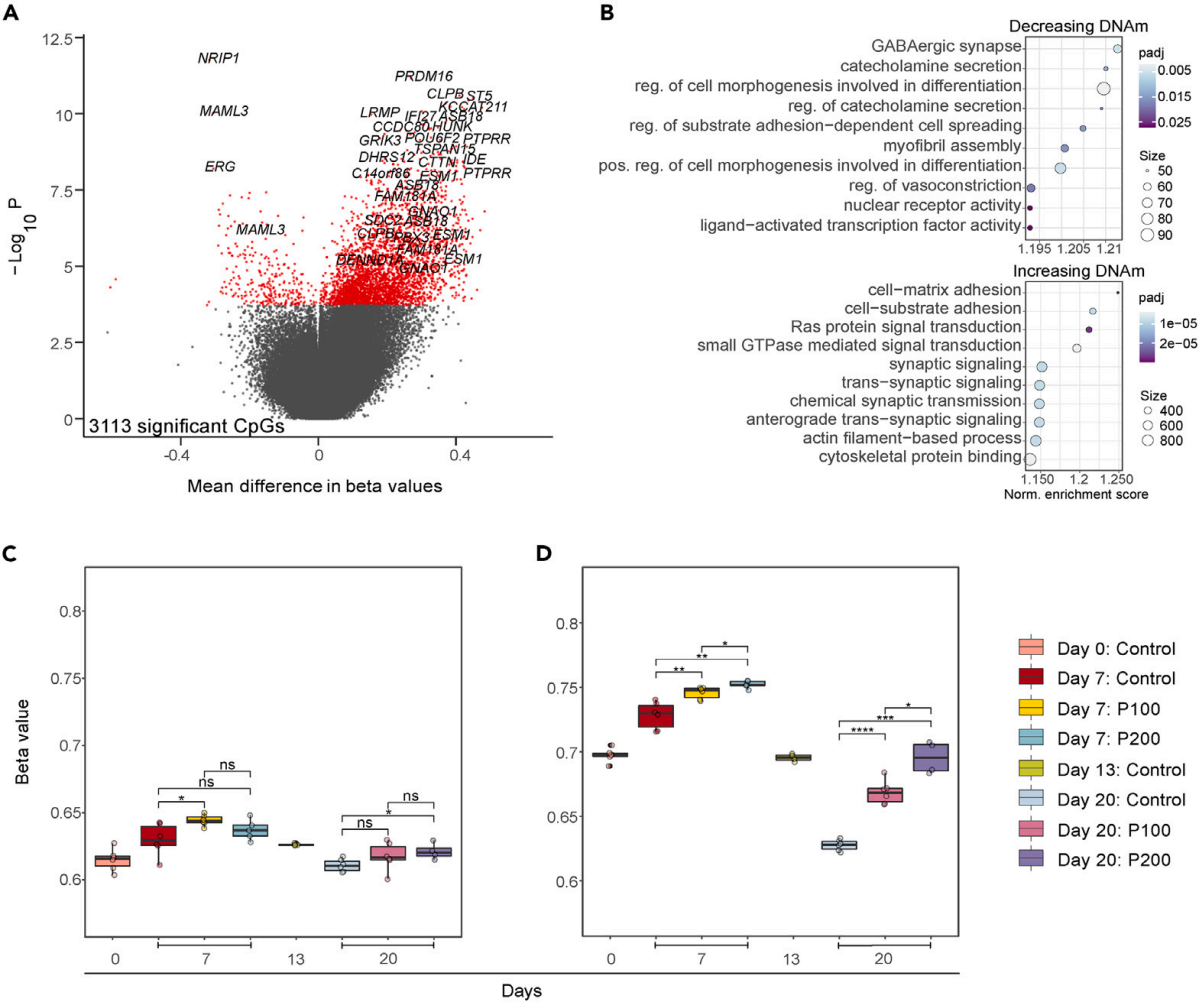
Exposure to paracetamol induces changes in DNAm over time during neuronal differentiation (A) Volcano plot showing the effect of 200 μM paracetamol from Day 7 to Day 20. CpGs with adjusted p value <0.05 were considered significant. (B) Corresponding enriched GO-terms for CpGs showing a decrease in DNAm (top) or an increase in DNAm (bottom) over time (Day 20 – Day 7) in P200 cells compared to control cells. Adjusted p-values are indicated by color. (C) Average DNAm levels per sample for all CpGs across differentiation for Day 0, 7, 13, and 20. (D) Average DNAm levels per sample for all significant CpGs (paracetamol-exposed cells vs. control comparisons) at Day 0, 7, 13, and 20. (C and D) ∗p < 0.05, ∗∗p < 0.01, ∗∗∗p < 0.001, ∗∗∗∗p < 0.0001.

To assess whether exposure of hESCs undergoing neuronal differentiation to paracetamol induced DNAm changes, we analyzed control cells and cells exposed to 100 (P100) and 200 μM paracetamol (P200) at Day 7 and 20. The experimental set-up included analysis of control cells harvested at Day 0 and 13 as reference points of possible dysregulation (Figures 5 and S7; Table S1). Assessment of principal component analysis showed clustering of samples according to differentiation day (Figure S7C; STAR Methods). Overall, the distribution of average global DNAm was indistinguishable across exposed and control cells at all time points regardless of paracetamol dose (Figure S7A). In contrast, non-CpG DNAm levels decreased during differentiation and were lower in cells exposed to 200 μM paracetamol compared to control cells at Day 20 (Figure S7B).

To investigate whether the cells exposed to different doses of paracetamol (P100 and P200) respond differently than control cells from Day 7 to Day 20, we performed a DNAm time-response analysis. We observed no significant DNAm changes in P100 compared to control between Day 7 and Day 20 (not shown). In contrast, 3 113 CpGs responded differently to P200 compared to control cells over time (Figure 5A). CpGs showing an increase in DNAm are annotated to genes with key functions in dynamic cellular redox changes in the developing brain, such as the neural specification gene *PRDM16*^66^ enriched for GO terms such as synaptic signaling and chemical synaptic transmission (Figure 5B). CpGs showing a decrease in DNAm are annotated to genes enriched for gene ontology (GO) terms involved in synaptic regulation, GABAergic signaling, and cell morphogenesis (Figure 5B). Overall, when we assessed DNAm levels at all CpGs (Figure 5C) compared to all significant CpGs (Figure 5D), we found a general dose-dependent increase in DNAm levels at significant sites in paracetamol-exposed cells compared to controls at both Day 7 and Day 20. The annotation of differentially methylated CpGs (DMCs) in relation to genes and CpG islands was similar across the different comparisons (Figures S7D and S7E).

**Figure 6:**
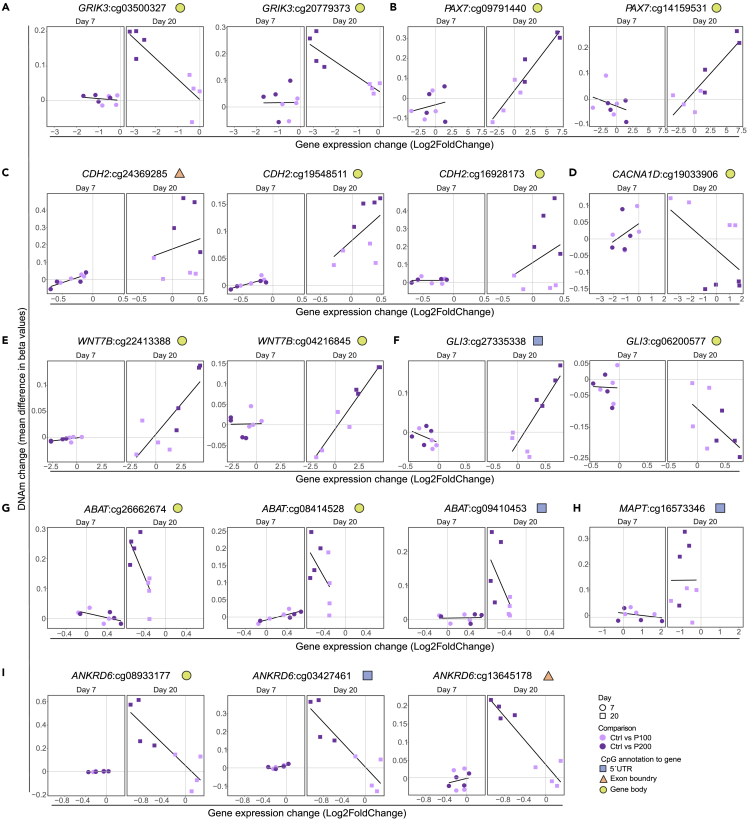
DNAm and gene expression for selected overlapping DMCs and DEGs (A–I) Change in DNAm and gene expression levels between control and P100 or P200 for (A) *GRIK3*, (B) *PAX7*, (C) *CDH2*, (D) *CACNA1D*, (E) *WNT7B*, (F) *GLI3*, (G) *ABAT*, (H) *MAPT* and (I) *ANKRD6*. Each point represents a matched replicate between RNA-seq and DNAm and black lines represents a linear regression line of the mean of values.

Next, we performed comparisons of DNAm levels in paracetamol-exposed cells to controls at Day 7 (Figures S7F and S7H; Table S1) and Day 20 (Figures S3G and S3I; Table S1) for the different doses. As expected, we observed more significant DNAm changes after longer exposure (Day 20) and at higher concentration of paracetamol (P200) (Table S1). We found no dose-dependent DMCs at Day 7 whereas at Day 20 a larger number of CpGs (n = 8 940) were differentially methylated between P100 and P200. Moreover, there was some overlap between DMCs at 7 and Day 20 (Figure S7J). To define a regulatory role of paracetamol induced DNAm changes on gene expression, we assessed the overlap between DEGs and differentially methylated genes (DMGs) for the pairwise comparisons and time-response analyses (Table S1). The percentage of DMGs overlapping with DEGs varied between 3 and 63% for the different comparisons. The P200 time-response analysis revealed 180 overlapping genes, and some selected genes are visualized in Figure 6. DNAm levels for *GRIK3*, *CACNA1D*, *ABAT*, *MAPT*, and *ANKRD6* were inversely correlated with gene expression, whereas DNAm levels for *PAX7*, *CDH2* and *WNT7B* were positively correlated with gene expression at Day 20. *GLI3* had DNAm levels that correlated with both negative and positive regulation (Figure 6).

### Overlap of dysregulated genes in differentiating hESCs and cord blood from children exposed to paracetamol during pregnancy

**Figure 7:**
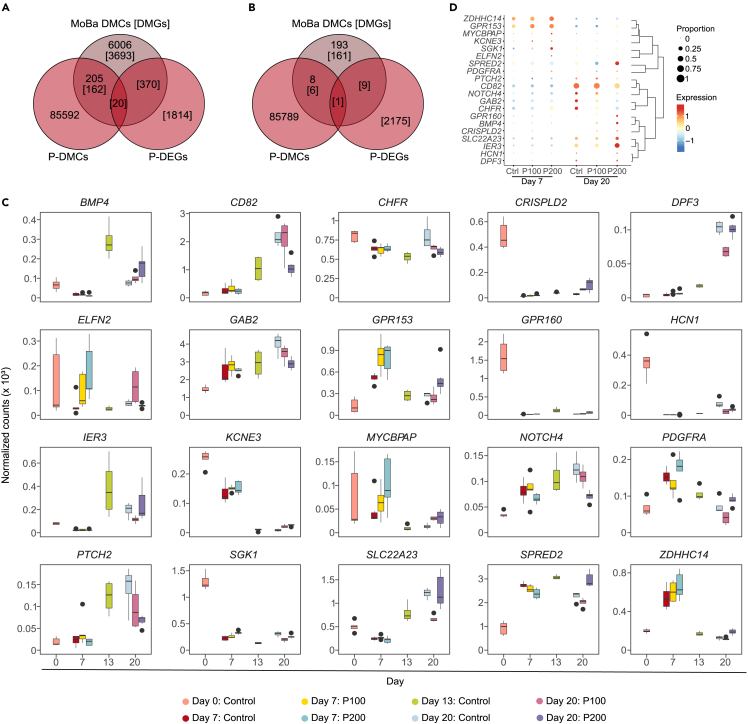
Overlap with differentially methylated genes in cord blood (A and B) Venn diagrams showing the overlap between Day 20 DEGs and DMCs for paracetamol comparisons and (A) paracetamol-exposed children with ADHD versus controls and (B) paracetamol-exposed children with ADHD versus ADHD controls in cord blood from MoBa. (C and D) Gene expression levels of overlapping genes identified in cord blood derived from (C) RNA-seq and (D) scRNA-seq data.

We have previously identified an association between differential DNAm in cord blood and long-term paracetamol exposure during pregnancy in children with ADHD.^20^ To assess the translational potential and causality of these findings, we compared the dysregulated genes identified in the present model to the DMGs associated with paracetamol exposure in cord blood. In brief, the DMCs and the DEGs in paracetamol-exposed differentiating cells at Day 20 were correlated with DMCs/DMGs in cord blood.^20^ Interestingly, we identified 20 genes that were both differentially methylated and differentially expressed in Day 20 paracetamol-exposed cells which overlapped with DMGs identified in cord blood between paracetamol-exposed children with ADHD versus controls (Figure 7A). Furthermore, and only one gene (*KCNE3*) overlapped between Day 20 DMCs, Day 20 DMGs and paracetamol-exposed children with ADHD versus ADHD controls in cord blood (Figure 7B).

We assessed the expression of these 20 overlapping genes in scRNA-seq data (Figure 7C) and RNA-seq data (Figure 7D). Notably, genes involved in differentiation, such as *GAB2*^67^*,* or *Notch and Hedgehog signaling pathways* (for example, *NOTCH4*,^68^ *PTCH2*,^69^ *SHISA2*^70^) show a dose-dependent downregulation of expression in paracetamol-exposed cells compared to controls. This high variation in expression levels after paracetamol exposure compared to controls, indicates a dose effect. Among genes identified in Day 7 P200 cells, we observed upregulation of *ZDHHC14*^71^ and *SGK1*,^72^ that are associated with control of neuronal excitability and neuronal response to injury, respectively. Several toxic insult response genes were also upregulated at Day 20 in the P200 treated cells, such as *IER3*,^73^ *SPRED2*,^74^ *GPR130*,^75^ and *SLC22A23*.^76^

## Discussion

Stem cell based systems for toxicological studies of medications allow for more realistic testing of concentrations *in vitro* and are often monitored using multi-omics approaches.^77^ Due to the inaccessibility of early human neurodevelopment for studies of the effects of paracetamol we have utilized a well characterized neuronally differentiating hESC protocol^30^ as an easily accessible and 3R’s compliant source. To our knowledge, this is the first multi-omics study investigating how paracetamol exposure affects epigenetic and transcriptional programmes using an *in vitro* hESC model of neuronal differentiation.^34^

We identified differential expression of genes enriched for BPs involved in *transmitter transport and regulation, synaptic organization and synaptogenesis and synaptic plasticity*. Interestingly, in cells exposed to the high dose of paracetamol (P200), we also identified enrichment of GO categories reflecting possible patterning deviations from forebrain differentiation. Thus, we observe an effect of paracetamol exposure on transcriptional dysregulation and possible developmental delays during neuronal differentiation. Additionally, among the common DEGs, irrespective of paracetamol dose, we identified *SORCS3,* which encodes a brain-expressed transmembrane receptor associated with neuronal development and plasticity that has been previously identified in a GWAS meta-analysis of ADHD significant risk loci.^78^ The scRNA-seq analysis identified dose-dependent DEGs involved in several major processes during neuronal differentiation. Exposure to paracetamol resulted in a subtle shift in cell populations from P1 to P2 at Day 7. At Day 20 the P6, P9 and P10 clusters decreased whereas P5 markedly increased in P200. The DEGs link paracetamol exposure to genes involved in cell-cycle length and phase transition and genes important for neuronal maturation, neurite outgrowth, cortical neurogenesis, expression of neurotransmitter transporters and WNT and FGF signaling. Further, the data also documented dose-dependent differential expression of crucial spatiotemporally regulated TFs associated with neuronal differentiation. These findings provide evidence of transcriptomic dose-dependent effects of paracetamol exposure related to cellular response to toxic insults and fate-determination deviation queues at Day 20. Furthermore, the results identified an impact of paracetamol exposure on the regulation of central, autonomic and sympathetic nervous system development, which are all central to finetuning human cognition and associated with ADHD.^79^^,^^80^^,^^81^

To further characterize and investigate epigenetic mechanisms involved in altered cellular heterogeneity and differentiation, we performed scATAC-seq to map CREs and compare the chromatin landscape changes between control and cells exposed to P100 and P200 at Day 20 (**hescneurodiffparacet**). Interestingly, the results from these analyses identified differences in several chromatin accessible regions in genes previously associated with fate mapping and neurogenesis, which have been identified in risk determination studies focusing on cognition and autism spectrum disorders (e.g., *DLK1, DIO3, IPW*^82^^,^^83^) and ADHD (e.g., *MEG3*^84^).

Genome-wide association studies (GWASs) suggest that cognitive disorders might also result from the cumulative effect of gene variant regulation and parent-of-origin effect,^84^ which in ADHD has been associated with genes such as *DDC, MAOA, PDLIM1*, and *TTR*. We also identified chromatin openness differences related to genes identified in the paracetamol exposed cells. For example, *PCDH7*, which acts as a cue for axonal guidance via roles in cell adhesion, was among the genes that were both differentially expressed and had different chromatin openness in Day 20 P200 cells. Dysregulation of *PCDH7* could be relevant to the semaphorin-plexin signaling documented by the enriched BPs in the P100 cells, and it is noteworthy as it has also been associated to ADHD.^78^

We also identified dose-dependent differences in chromatin accessibility of the neural specification gene locus *PRDM16*, which correlates well with DNAm analyses where exposure to paracetamol was associated with increased DNAm at CpGs annotated to *PRDM16*. This gene has been linked to regulation of transcriptional enhancers activating genes involved in intermediate progenitor cell production and repressing genes involved in cell migration.^66^ The molecular mechanism of *PRDM16* remains unknown but has been associated with reactive oxygen species regulation in the embryonic cortex.^85^

Integrative analyses of scRNA-seq and scATAC-seq to explore the links between chromatin accessibility and gene expression documented high correlation and showed an overlap between scATAC-seq and scRNA-seq cell clusters. This analysis identified many putative CREs, and linked genes affected by paracetamol exposure. Although a portion of linked genes overlapped between the control and exposed cells, there was only a small overlap between the putative CREs. This suggest that many local changes in chromatin opening were affected by paracetamol exposure and that these CREs may regulate different gene expression programmes in control and paracetamol exposed cells. How paracetamol regulates chromatin opening is not known, and we have limited knowledge on the effect of paracetamol on the TFs recognition of DNA motifs. However, our data suggest that genes linked to putative CREs that were affected by paracetamol exposure have a role in regulation of transcription and chromatin. In particular, a linked gene specific for paracetamol exposure was *EP300*, which codes for a histone acetyltransferase responsible for the active enhancer mark H3K27ac.^86^ *EP300* expression decreased subtly upon paracetamol exposure both at Day 7 and 20, and these changes in EP300 levels may change the number of active enhancers in the paracetamol exposed cells. Furthermore, we identified TFs such as EMX2, TBR1 and OTX2 whose regulatory activity is affected by paracetamol exposure at the differentiation endpoint. This is suggestive of a mechanism for how paracetamol exposure may change gene expression programmes in early brain development. More work is needed to follow up the functional role of paracetamol exposure on the enhancer regulation and gene expression.

We found a general dose-dependent increase in DNAm in paracetamol exposed cells compared to controls at both Day 7 and Day 20. The genes were enriched for BPs involved in cell morphogenesis and adhesion, but also synaptic transmission and signaling with a focus on catecholamines and GABAergic transmission. Considering that this is an *in vitro* system, the findings are compelling since structural cortical changes and catecholaminergic transmission have been the target of various epidemiological studies and drug clinical trials, both in children and adults with ADHD.^87^^,^^88^^,^^89^^,^^90^ It is biologically plausible that medications that cross the placenta and the blood-brain barrier, may interfere with normal fetal brain neurodevelopment, previously shown for valproic acid, and more recently for topiramate.^91^ This is especially relevant for substances that cross the blood-brain barrier, which is considered functional by the 8th week of gestation.^92^ However, invasive testing of the fetal compartment in human is rarely indicated, which makes data on safety during pregnancy for most substances scant. Thus, most pharmacoepigenetic studies address associations of DNAm differences in cord blood or placenta from neonates delivered at term exposed to the maternal medication during pregnancy, which is also the case for paracetamol.^93^^,^^94^^,^^95^

We previously found DMGs in cord blood from children with ADHD exposed to long-term maternal paracetamol use.^20^ However, the causality, and relevance of such findings to brain development are not known. The hESCs used in our experiments represent the inner cell mass (ICM) of Day 6 preimplantation-blastocysts.^96^ The ICM is known to differentiate to form the primitive ectoderm around Day 7.5 post fertilization,^97^ but placental circulation is not established before Day 17–20.^98^ Prior to this, the fraction of paracetamol that reaches the developing embryo does so via passive diffusion.^99^ Interestingly, irrespective of the temporal differences of the two models,^100^ comparing the differentially expressed and methylated genes identified in our study revealed overlap of several genes identified in cord blood with potential compelling relevance to early brain development. Notably, identification of a dose-specific effect on several genes have previously been shown to be associated with neural injury and toxic-insult response.^101^^,^^102^^,^^103^ To our knowledge this is the first study that has identified an effect of paracetamol on differential DNAm of *KCNE3* in a neuronal differentiation model of hESCs. Differential DNAm at *KCNE3* was also identified in our MoBa study in cord blood.^20^ Furthermore, *KCNE3* was also identified in a DNAm analysis of extremely low gestational age new born (ELGAN) cohort.^93^ *KCNE3* is an interesting candidate, which encodes a voltage-gated ion channel with important functions in regulating release of neurotransmitters and neuronal excitability.^104^^,^^105^ Among the overlapping genes, there was also *IER3*, whose differential DNA methylation at specific CpG sites has been previously associated with *in utero* exposure to bisphenol A (BPA).^106^ The same study had identified *TSPAN15*, which is also significantly differentially methylated between control and P200 cells in our neuronal differentiation model.

We also compared our results to previously published studies by exploring expression of relevant genes in the webtool (**hescneuroparacet**). In a rat model of fetal paracetamol exposure,^107^ dysregulation in specific ABC efflux transporters and related enzymes was found after chronic treatment of the mothers in E19 fetal brain and choroid plexus. In our model of paracetamol exposure, we also identified differential expression of the relevant genes *ABCA1, ABCC5, ABCD3*, and *GSTM*3. Moreover, the expression of *ABCC4*, which encodes the multidrug resistance-associated protein MPRP4, known to play a role in paracetamol efflux was also upregulated at Day 20.^108^ In another model assessing chronic paracetamol exposure effects in E15-19 rat brains,^109^ similar to our findings, an increased expression of genes related to proliferation (e.g., *MKI67*, *MTDH*), cytoskeletal structural genes (e.g., *NEFL* at Day 20), metabolic stress alleviation (e.g., *PFKP* at Day 7) or decrease in genes related to differentiation (*SOX12* at Day 7) migration and dendrite orientation (such as *CRMP1* at Day 20) was found. Notably, a significant increase in *APOE* expression at P200 cells at Day 7 was observed indicating stress or neuronal damage,^110^ and a similar response by the increased expression for glutamate transporter *EAAT4*.^111^ A DNA repair gene, *RAD54L*, was upregulated in P200 treated cells at Day 7 (Table S2C), and interestingly this gene was also found differentially expressed in the prefrontal cortex of paracetamol-exposed mice.^112^ Also, a stress response transcription factor *ATF4* was found significantly downregulated in P200 cells at Day 7 (Table S2C) and has been shown to be differentially expressed in a single-cell study of mice hepatocytes upon paracetamol overdose.^113^ Moreover, the significant *IL6ST* (GP130) and *MEF2A*^114^ upregulation and the similarities of our findings to the other *in vivo* models, validate both the trajectories identified by our approach, and the chosen paracetamol neurotoxicity platform for early development studies.

Abiding by the Findability, Accessibility, Interoperability, and Reusability (FAIR) principles, we provide full access to the scRNA-seq and scATAC-seq datasets in open access shiny web platforms for scRNA-seq (**hescneuroparacet**) and integrative scATAC-seq/scRNA-seq (**hescneurodiffparacet**).^115^^,^^116^ These webtools allow for data correlation with other published gene expression datasets, and enable plotting, exporting, and downloading high resolution figures of gene expression and gene co-expression analysis UMAPs of any gene. Furthermore, there are multiple options for dataset exploration and visualization, such as heatmaps, violin-, box-, proportion- and bubble plots in different tabs, where gene expression may be viewed per cell, cluster, treatment and timepoint. Chromatin opening can be explored for genomic loci of interest for input sample, ATAC clusters or integrated clusters in a genome browser view with peak-to-gene link annotations (instantly plotted for 500 random cells each time). In addition, the processed datasets and code are made available for customization and integration with other studies. To our knowledge this is the first *in vitro* study of paracetamol exposure of hESCs under neuronal differentiation, that provides access to scRNA-seq and scATAC-seq data via interactive webtools.

### Limitations of the study

Our study has several limitations and strengths. First, paracetamol is metabolized into inactive glucuronide and sulfate conjugates and the highly reactive metabolite, NAPQI, in the liver, but the presence of the required enzymes, transporters and breakdown molecules, were not measured in this study. Future studies are needed to characterize paracetamol metabolism in hESCs and potential effect of paracetamol conjugates and metabolites on neuronal differentiation. Moreover, the protein expression changes, and enzyme activities were not explored, as this was beyond the scope of this study. This study aimed to delineate the epigenetic impact of paracetamol on early human cortical developmental events. We used both bulk- and scRNA-seq and comparing these two datasets identified a varying degree of overlapping DEGs from 1 to 27%, confirming that both datasets are complementary to detect transcriptomic changes induced by paracetamol. Using a multi-omics approach, we unveiled the diversified transcriptional networks related to paracetamol exposure. We could not accurately classify the maturation properties of the cells, or the proportion of cells in G0 phase, highlighting the necessity of new tools to deconvolute the neuronal G0 phase. The results of scATAC-seq analysis presented here are inherently limited by the relatively low genome-per-cell coverage. That means that some open chromatin regions that could have proved relevant for the individual cells or cell populations, may have been missed. Finally, the *in vitro* results presented here need to be validated by *in vivo* models and targeted human dataset exploration in future studies. This could confirm whether the pathways identified can explain the paracetamol-induced adverse effects in the early human brain.

In conclusion, using an *in vitro* hESC model of neuronal development, we identified altered gene expression, DNAm and chromatin openness involved in key neuronal differentiation processes at bulk and single-cell RNA levels. An overlap of genes identified in this model and in the cord blood of neonates exposed long-term to paracetamol during pregnancy, points toward altered epigenetic regulation of early brain development. Identification of common DNAm modification sites and chromatin openness regions with a regulatory role on gene expression can identify loci and underlying mechanisms involved in neurotoxicity of drugs on the developing fetus with potential effect on long-term outcomes. Such findings could strengthen causal inference and clinical translation of altered DNAm, such as in cord blood, on brain development. However, these *in vitro* results need further validation for potential clinical translation.

## STAR★Methods

### Key resources table

**Table:** 

REAGENT or RESOURCE	SOURCE	IDENTIFIER
**Chemicals, peptides, and recombinant proteins**		

Geltrex™ LDEV-Free, hESC-Qualified, Reduced Growth Factor Basement Membrane Matrix	ThermoFisher	A1413302
KnockOut™ DMEM	ThermoFisher	10829018
PBS, no calcium, no magnesium	ThermoFisher/GIBCO	14190
Dimethyl -sulfoxide, DMSO	Sigma-Aldrich/ Merck	D8418
Accutase™ Cell Detachment Solution	STEMCELL Technologies	7920
UltraPure 0.5 M EDTA, pH 8.0	ThermoFisher	15575020
Paracetamol	Sigma-Aldrich/Merck	A7085
RHO/ROCK Pathway Inhibitor Y-27632	STEMCELL Technologies	SCM075
Essential 8™ Medium	ThermoFisher	A1517001
Poly-L-ornithine hydrobromide	Sigma-Aldrich/ Merck	P3655
Fibronectin (Bovine Protein, Plasma)	ThermoFisher	33010018
N2 supplement (100X)	ThermoFisher	17502048
Advanced DMEM/F-12	ThermoFisher	12634028
GlutaMAX™ Supplement	GIBCO/ ThermoFisher	35050061
Penicillin Streptomycin (10,000 U/mL)	ThermoFisher	15140122
LDN-193189	STEMCELL Technologies	72148
SB 431542 (hydrate)	Sigma-Aldrich / Merck	S4317
XAV939	STEMCELL Technologies	72674
B-27™ Supplement (50X), serum free	ThermoFisher	17504044
Recombinant Human FGF basic	Peprotech	100-18B
Recombinant Human EGF, Animal-Free	Peprotech	AF-100-15

**Critical commercial assays**		

Countess™ Cell Counting Chamber Slides	ThermoFisher	C10312
RNeasy Mini Kit	Qiagen	74106
RNAse-Free DNase Set	Qiagen	79254
RNA/DNA purification kit	Norgen Biotek Corp.	298-48700
RNase-Free DNase I Kit	Norgen Biotek Corp.	298-25720
Qubit™ RNA BR Assay Kit	ThermoFisher/Invitrogen	Q10211
QuantiTect Reverse Transcription Kit	Qiagen	205311
ddPCR Supermix for Probes (no dUTP)	BioRad	186-3024
Droplet Generation Oil for Probes	BioRad	186-3005
TruSeq Stranded mRNA Library Prep Kit	Illumina	20020595
IDT for Illumina – TruSeq RNA UD Indexes	Illumina	20022371
NovaSeq 6000 S1 Reagent Kit v1.5 (200 cycles)	Illumina	20028318
Infinium MethylationEPIC BeadChip Kit (96 samples)	Illumina	WG-317-1003
30 mm MACS SmartStrainers	Miltenyi Biotech	130-110-915
Chromium Single Cell 3′ Library & Gel Bead Kit v3	10x Genomics	1000075
Chromium i7 Multiplex Kit	10x Genomics	120262
NextSeq 500/550 High Output Kit (150 Cycles)	Illumina	20024907
Next GEM Chip H Single Cell Kit	10x Genomics	1000162
Next GEM Single Cell ATAC Library & Gel Bead Kit v1.1	10x Genomics	1000176
Chromium i7 Multiplex Kit N Set A	10x Genomics	1000084
NovaSeq 6000 SP Reagent Kit (100 cycles)	Illumina	20028401

**Deposited data**		

RNA-seq, DNAm, Infinium Methylation EPIC, scRNA-seq & scATAC-seq	This paper	NCBI GEO: GSE220027 (Subseries GSE220023, GSE220024, GSE220025, GSE220026)
RNA-seq, DNAm, Infinium Methylation EPIC, scRNA-seq & scATAC-seq	Samara et al.^34^	NCBI GEO: GSE192858 (Subseries GSE192854, GSE192855, GSE192856, GSE192857)

**Experimental models: Cell lines**		

Human embryonic cells, HS360, 46XY.	Stockholms Medicinska Biobank / Sweden	HS360

**Oligonucleotides**		

*POU5F1*	ThermoFisher/TaqMan™	Hs00999632_g1
*SOX2*	ThermoFisher/TaqMan™	Hs01053049_s1
*NANOG*	ThermoFisher/TaqMan™	Hs04399610_g1
*NES*	ThermoFisher/TaqMan™	Hs04187831_g1
*FOXG1*	ThermoFisher/TaqMan™	Hs01850784_s1
*TUBB3*	ThermoFisher/TaqMan™	Hs00801390_s1
*MAP2*	ThermoFisher/TaqMan™	Hs00258900_m1
*PAX6*	ThermoFisher/TaqMan™	Hs00240871_m1
*OTX2*	ThermoFisher/TaqMan™	Hs00222238_m1
*VIM*	ThermoFisher/TaqMan™	Hs00958111_m1
*NEUROD1*	ThermoFisher/TaqMan™	Hs01922995_s1
*RPL30*	ThermoFisher/TaqMan™	Hs00265497_m1
*RAF1* F: tgggaaatagaagccagtgaa R: cctttaggatctttactgcaacatc	Eurofins	Probe 56 4688538001

**Software and algorithms**		

R Programming language	R Core Team^117^	https://www.r-project.org/
ArchR1.0.1	Granja et al.^62^	https://www.archrproject.com
Seurat Version 4	Hao et al.; Stuart et al.^118^^,^^119^	https://github.com/satijalab/seurat
Signac	Stuart et al.^120^	https://satijalab.org/signac/
BSgenome1.58.0	Pages^121^	https://rdrr.io/bioc/BSgenome/
ShinyCell	Ouyang et al.^122^	https://github.com/SGDDNB/ShinyCell
ShinyArchR.UiO	Sharma et al.^116^	https://github.com/EskelandLab/ShinyArchRUiO
CytoTRACE R package (v0.3.3)	Gulati et al.^123^	https://cytotrace.stanford.edu
10x Genomics Cell Ranger -Count and 10x Genomics Cell Ranger -Count ATAC	10x genomics	https://www.10xgenomics.com
BSgenome.Hsapiens.UCSC.hg38	https://doi.org/10.18129/B9.bioc.BSgenome.Hsapiens.UCSC.hg38 (The Bioconductor Dev Team, 2021)^124^	https://bioconductor.org/packages/release/data/annotation/html/BSgenome.Hsapiens.UCSC.hg38.html
EnsDb.Hsapiens.v86	https://doi.org/10.18129/B9.bioc.EnsDb.Hsapiens.v86 (Rainer, 2017)^125^	https://bioconductor.org/packages/release/data/annotation/html/EnsDb.Hsapiens.v86.html
clustree	Zappia and Oshlack^126^	https://cran.r-project.org/web/packages/clustree/vignettes/clustree.html#references
scater	McCarthy et al.^127^	https://bioconductor.org/packages/release/bioc/html/scater.html
DEseq2	Love et al.^128^	https://bioconductor.org/packages/release/bioc/html/DESeq2.html
GSEA	Subramanian et al.^129^	https://www.gsea-msigdb.org/gsea/index.jsp
Minfi	Aryee et al.^130^	https://www.bioconductor.org/packages/release/bioc/html/minfi.html
Limma	Ritchie et al.^131^	https://bioconductor.org/packages/release/bioc/html/limma.html
methylGSA	Ren and Kuan et al.^132^	https://bioconductor.org/packages/release/bioc/html/methylGSA.html
SingleR	Aran et al.^133^	https://github.com/dviraran/SingleR and http://bioconductor.org/books/release/SingleRBook/sc-mode.html
Harmony	Korsunsky et al.^134^	https://github.com/immunogenomics/harmony
GREAT	McLean et al.^135^	http://great.stanford.edu/public/html/
Intervene	Khan and Mathelier^136^	https://github.com/asntech/intervene-shiny
g:Profiler	Kolberg et al.; Raudvere et al.^35^^,^^137^	https://biit.cs.ut.ee/gprofiler/gost
MACS2	Feng et al.^138^	https://pypi.org/project/MACS2/ https://github.com/macs3-project/MACS
refdata-cellranger-atac-hg38-version refdata-	10x genomics	https://support.10xgenomics.com/single-cell-atac/software/downloads/
JASPAR	Bryne et al.^139^	https://bioconductor.org/packages/release/data/annotation/html/JASPAR2020.html http://jaspar.genereg.net
Single Cell Experiment	Amezquita et al.^140^	https://bioconductor.org/packages/release/bioc/html/SingleCellExperiment.html
Escape	https://doi.org/10.18129/B9.bioc.escape135^141^	https://github.com/ncborcherding/escape
ggseqlogo	Waight^142^	https://CRAN.R-project.org/package=ggseqlogo
TFBSTools	https://doi.org/10.18129/B9.bioc.TFBSTools	https://bioconductor.org/packages/release/bioc/html/TFBSTools.html
uwot	James Melville (https://github.com/jlmelville/uwot)	https://CRAN.R-project.org/package=uwot
viridisLite	Garnier et al.^143^	https://cran.r-project.org/web/packages/viridisLite/index.html
ShinyGO	Ge et al.^144^	http://bioinformatics.sdstate.edu/go/
Python3	Van Rossum and Drake^145^	https://www.python.org/downloads/
10x Genomics Loupe Browser	10x genomics	https://www.10xgenomics.com
ComplexHeatmap	Gu; Gu et al.^146^^,^^147^	https://jokergoo.github.io/ComplexHeatmap-reference/book/
ggplot2	Wickham^148^	https://cran.r-project.org/web/packages/ggplot2/index.html
igraph	igraph	https://igraph.org
IRanges	Lawrence et al.^149^	https://bioconductor.org/packages/release/bioc/html/IRanges.html
Reticulate	Tomasz Kalinowski, R Studio	https://CRAN.R-project.org/package=reticulate
tidyverse	Wickham et al.^150^	https://www.tidyverse.org/packages/
ggpubr	Kassambara^151^	https://cran.r-project.org/web/packages/ggpubr/index.html
SARTools	Varet et al.^152^	https://github.com/PF2-pasteur-fr/SARTools
pheatmap	Raivo Kolde (https://github.com/raivokolde/pheatmap)	https://CRAN.R-project.org/package=pheatmap
lluminaHumanMethylationEPICmanifest	Hansen^153^	https://bioconductor.org/packages/release/data/annotation/html/IlluminaHumanMethylationEPICmanifest.html
IlluminaHumanMethylationEPICanno.ilm10b5.hg38	EPIC annotation 1.0 B5	https://github.com/achilleasNP/IlluminaHumanMethylationEPICanno.ilm10b5.hg38
Rstudio	RStudio Team	https://www.rstudio.com/
Propeller	Phipson et al.^53^	https://github.com/phipsonlab/speckle
Shiny single-cell tools for visualisation of datasets.	This paper	Custom scripts for computational analysis are available athttps://github.com/EskelandLab/Neuralparacet. The single-cell data can be explored in webtools for scRNA-seq (**hescneuroparacet**) and integrative scATAC-seq/scRNA-seq (**hescneurodiffparacet**) at https://cancell.medisin.uio.no/.

### Resource availability

#### Lead contact

Further information and requests for resources and reagents should be directed to and will be fulfilled by the lead contact Ragnhild Eskeland (Ragnhild.Eskeland@medisin.uio.no).

#### Materials availability

This study did not generate new unique reagents.

### Experimental model and study participant details

#### hESC culture and maintenance

hESCs HS360 (Karolinska Institutet, Sweden, RRID:CVCL C202)^96^^,^^154^ were cultured as described by Samara et al., 2022.^30^ In brief, cells were maintained in Essential 8™ Medium, on Geltrex pre-coated culture plates. Cells were routinely passaged at 75-85% confluency using 0.5 mM ethylenediaminetetraacetic acid (EDTA) in ratios between 1:3 to 1:6. When hESCs were collected for initiation of the differentiation protocol and Accutase was used to detach and dissociate cells. Mycoplasma tests were routinely performed.

#### Neuronal differentiation of hESCs and exposure to paracetamol

hESCs HS360 were differentiated, as described,^30^ in two separate time-course experiments, to investigate the *in vitro* effects of paracetamol exposure in physiologically relevant concentrations to long-term exposure *in vivo*. Since paracetamol crosses the placenta and the blood-brain barrier and has an insignificant plasma protein binding, maternal plasma/serum and cord blood concentrations can be used as estimates for the amount of paracetamol that reaches the developing fetal brain.^155^^,^^156^^,^^157^^,^^158^ Physiological plasma peak concentration range of paracetamol *in vivo* were determined to be 100 μM and 200 μM, respectively, corresponding to one intermediate and one high peak plasma concentration.^31^^,^^32^^,^^33^ Thus, cells undergoing neuronal differentiation were exposed to changes of medium only (control) or medium containing 100 or 200 μM paracetamol from Day 1 to Day 20. Control cells were harvested at day 0, 7, 13 and 20, while cells exposed to 100 or 200 μM paracetamol were harvested at Day 7 and 20 (Table 1).

### Method details

#### DNA/RNA isolation

Genomic DNA and total RNA were isolated by direct lysis in the culture vessels followed by column-based isolation using RNA/DNA purification kit (Norgen Biotek). RNase-Free DNase I Kit (Norgen Biotek) was applied for on-column removal of genomic DNA contamination from RNA isolates. Five RNA isolates were processed using the RNeasy Mini Kit (Qiagen) followed by DNase-treatment using the RNAse-Free DNase Set (Qiagen). All isolations were performed according to the manufacturer's instructions. Nucleic acid quantification was performed using Qubit (ThermoFisher Scientific), purity was measured using Nanodrop 2000 (ThermoFisher Scientific), while RNA and DNA integrity was assessed using 2100 Bioanalyzer (Agilent Technologies) and 4200 TapeStation (Agilent Technologies), respectively.

#### ddPCR and RNA expression analysis

Reverse transcription of total RNA was performed using QuantiTect Reverse Transcription Kit (Qiagen). Subsequent ddPCR reactions were set up using ddPCR Supermix for Probes (No dUTP) (BioRad) and Taqman assays (ThermoFisher) or Universal Probes (Roche) in combination with target primers (Eurofins) as outlined in key resources table. Droplets for ddPCR amplification were generated using the QX200 Droplet Generator (BioRad). Data acquisition and primary analysis was done using the QX200 Droplet Reader (BioRad) and QuantaSoft software (BioRad). All steps were performed according to the manufacturer's instructions. To calculate the number of target copies per ng RNA input, samples were normalized using RPL30 and RAF1 as normalization genes.^159^ Statistical comparisons were performed in R using t-test in ggpubr package v.0.4.0.^151^ Results were visualized in R using the tidyverse package.^150^

#### Bulk RNA-seq

The sequencing library was prepared with TruSeq Stranded mRNA Library Prep (Illumina) according to manufacturer’s instructions. The libraries (n=39) were pooled at equimolar concentrations and sequenced on an Illumina NovaSeq 6000 S1 flow cell (Illumina) with 100 bp paired end reads. The quality of sequencing reads was assessed using BBMap v.34.56^160^ and adapter sequences and low-quality reads were removed. The sequencing reads were then mapped to the GRCh38.p5 index (release 83) using HISAT2 v.2.1.0.^161^ Mapped paired end reads were counted to protein coding genes using featureCounts v.1.4.6-p1.^162^ Differential expression analysis was conducted in R version 3.5.1^163^ using SARTools v.1.6.8^152^ and the DESeq2 v.1.22.1.^128^ For the time-response analysis, the time effect of 100 or 200 μM paracetamol compared to control (no paracetamol) from Day 7 to Day 20 (Day 7 as reference), was modelled using the following design: “∼ Concentration + Day + Concentration:Day” using the interaction term to find any treatment-specific differences over time. Genes were considered significantly differentially expressed with an FDR < 0.05. Normalized counts were visualized using the tidyverse package v.1.3.^150^ The heatmaps were generated using the pheatmap package version 1.0.12.^164^ The Wald-test was used to calculate p-values and Benjamini-Hochberg was used to correct for multiple testing. The Gene Set Enrichment Analysis (GSEA) analysis was performed using pre-ranked gene lists, based on p-values and direction of expression change by a competitive test, into GSEA software v.4.1.0^129^ to identify enrichment of BP terms. The size of the analysed gene sets was restricted to 15-1000 genes, and the chip annotation used was “Human_ENSEMBL_Gene_ID_MSigDB.v7.2.chip”. Moreover, we performed analysis of BPs on differentially expressed genes from cells exposed to 100 or 200 μM paracetamol compared to control cells over time (Day 7 - Day 20) (Tables S1H and S1I) analysed using a server based g:Profiler^53^ showing results with correlation greater than 0.45_significant.

#### Bulk DNAm analysis

DNAm status of 43 samples were assessed using the Infinium MethylationEPIC BeadChip v.1.0_B3 (Illumina). Quality control and pre-processing of the raw data was performed in R using Minfi v.1.36.0.^130^ No samples were removed due to poor quality (detection p values >0.05). Background correction was performed using NOOB method^165^ and β values (ratio of methylated signal divided by the sum of the methylated and unmethylated signal) were normalized using functional normalization.^166^ Probes with unreliable measurements (detection p values >0.01) (n = 12,538) and cross-reactive probes (45) (n = 42,844) were then removed, resulting in a final dataset consisting of 810,477 probes and 43 samples. Probes were annotated with Illumina Human Methylation EPIC annotation 1.0 B5 (hg38). Differential DNAm analysis for pairwise and time-response comparisons was performed on the M values (log2 of the β values) using the limma package^131^ with the following design: “∼0+group”, where the group was Day + Treatment combined. The contrast matrix specified time-response comparisons the following way:

“TR = (PX_Day20 – PX_Day7) -(Control_Day20 - Control_Day7)” , where PX is either 100 or 200 μM paracetamol. CpGs were considered significantly differentially methylated with an FDR < 0.05. GO analysis of BP terms was performed using p-values of increasing or decreasing CpGs (DMCs) as input to methylRRA function implemented in the methylGSA package version 1.14.0.^132^

#### Collection of cells and scRNA-seq

Cells were washed twice in wells with 1x PBS and detached using Accutase (STEMCELL Technologies) at 37°C for 7 min. Cells were triturated 10-15 times to separate into single cells and transferred to centrifuge tubes containing the appropriate base media with 0.05 % BSA (Sigma-Aldrich). Counts were performed using Countess II FL Cell Counter (ThermoFisher Scientific) before cells were centrifuged at 300x g for 5 min and the supernatant was discarded. Cell pellets were then resuspended in base medium containing 0.05 % BSA and cell aggregates were filtered out using MACS SmartStrainers (Miltenyi). The cells were recounted and processed within 1 hour on the 10x Chromium controller (10x Genomics). Approximately 2,300 cells were loaded per channel on the Chromium Chip B (10x Genomics) to give an estimated recovery of 1,400 cells. The Chromium Single Cell 3′ Library & Gel Bead Kit v3 (10x Genomics) and Chromium i7 Multiplex Kit (10x Genomics) were used to generate scRNA-seq libraries, according to the manufacturer's instructions. Libraries from 16 samples were pooled together based on molarity and sequenced on a NextSeq 550 (Illumina) with 28 cycles for read 1, 8 cycles for the I7 index and 91 cycles for read 2. For the second sequencing run, libraries were pooled again based on the number of recovered cells to give a similar number of reads per cell for each sample (33,000 - 44,000 reads/cell).

#### scRNA-seq data analysis

The Cell Ranger 3.1.0 Gene Expression pipeline (10x Genomics) was used to demultiplex the raw base-call files and convert them into FASTQ files. The FASTQ files were aligned to the GRCh38 human reference genome, and the Cell Ranger Count command quantified single-cell read counts using default parameters for Days 7 and 20. Cell ranger aggregate was used for aggregating counts of replicates. The Seurat Package v.4.1.0^119^ was used to perform quality control and normalization on the count matrices. We filtered cells with number of RNA counts >= 2000 & <= 50000 to remove dead cells, doublets and multiplets. The cells expressing fewer genes (less than 200) and genes expressed in less than 3 cells were excluded from the downstream analysis. Outlier cells with high Mitochondrial percentage was computed using Scater package 1.0.4 “isoutlier” function with nmads parameter of 5.^127^ Counts were adjusted for cell-specific sampling using the SCTransform function with regression of Cell cycle genes and Mitochondrial content.^167^^,^^168^ We used a resolution of 0.4 to cluster cells, obtained by determining the optimum number of clusters (cells grouped together sharing similar expression profiles) in the dataset using the Clustree R package.^126^ Principal component analysis was performed using the RunPCA function, followed by FindClusters and RunUMAP functions of Seurat package to perform SNN-based UMAP clustering. We used Propeller^53^ and plotCellTypeProps functions of Propeller package to compute differences in cell cluster proportions using default logit transformation of proportions. One-way analysis of variance (ANOVA) was performed for all statistical tests on different groups i.e., paracetamol exposure at Day 7 and Day 20 versus control Day 7 and Day 20.

FindMarkers from the Seurat R package were used to perform differential expression analysis between groups. For DE between exposure groups, thresholds were set to the following: min.pct = 0.25, min.diff.pct = -Inf, logfc.threshold = 0.1. For top overlapping DE genes per Day (Figures 6E and 6I), thresholds were set to the following: min.pct = 0.25, min.diff.pct = -Inf, logfc.threshold = 0.25. Genes with an adjusted p-value < 0.05 were considered significant. GO analysis was performed using the DEenrichRPlot function of the mixscape R package with the "GO_Biological_Process_2018'' database with the following thresholds: logfc.threshold = 0.25, max genes = 500. The SingleR R package^133^ was used to annotate the cells against reference data sets from an Early Human Brain dataset (hESDB)^54^ from the scRNAseq R package.^169^ Cell types with < 15 cells annotated were excluded from the UMAP plot (Figure 3F). Four full singleR plots using reference dataset for annotation relevant for neuronal cell types can be viewed in the webtool (**hescneuroparacet**) in the tab cell informations: SingleR.hESCs, hEMBs and hESBD (single-cell data from neuronally differentiating embryonic stem cells, early human ventral midbrain or combined respectively)^54^ and SingleR.HPCA (Human Primary Cell Atlas, from microarray samples, 38 main cell types and 169 subtypes).^133^ Cell number/statistics can be viewed by pressing the toggle button.

#### scATAC-seq library preparation and sequencing

Cells were washed twice with 1xPBS and detached to single cell suspension by application of Accutase (STEMCELL Technologies) at 37°C for 7 min. The detached cells were washed with appropriate base media with added 0.04% BSA (Sigma-Aldrich) and filtered using MACS SmartStrainers (Miltenyi Biotech) to remove cell aggregates. Nuclei isolation was done according to the 10x Genomics protocol CG000169 (Rev D) using 2 minutes of incubation in a lysis buffer diluted to 0.1x and 0.5x for Day 0 and Day 20 cells, respectively. Countess II FL Cell Counter (ThermoFisher Scientific) was used to quantify nuclei and confirm complete lysis and microscopy to confirm high nuclei quality. Nuclei were further processed on the 10x Chromium controller (10x Genomics) using Next GEM Chip H Single Cell Kit (10x Genomics), Next GEM Single Cell ATAC Library & Gel Bead Kit v1.1 (10 x Genomics) and Chromium i7 Multiplex Kit N Set A (10x Genomics) according to the Next GEM Single Cell ATAC Reagent Kits v1.1 User Guide (CG000209, Rev C). The targeted nuclei recovery was 5,000 nuclei per sample. The resulting 4 sample libraries were sequenced on a NovaSeq Sp flow cell (Illumina) with 50 cycles for read 1, 8 cycles for the i7 index read, 16 cycles for the i5 index read and 49 cycles for read 2.

#### scATAC sequencing analysis

Cell Ranger ATAC version 1.2.0 with reference genome GRCh38-1.2.0 was used to pre-process scATAC-seq raw sequencing data into FASTQ files. Single-cell accessibility counts for the cells were generated from reads using the cellranger-atac count pipeline. Reference genome HG38 used for alignment and generation of single-cell accessibility counts was obtained from the 10x Genomics (https://support.10xgenomics.com/single-cell-atac/software/downloads/). Downstream analysis of the scATAC-seq data was performed using the R package ArchR v1.0.1.^62^ A tile matrix of 500-bp bins was constructed after quality control, removal of low-quality cells and doublet removal using the doubletfinder function of ArchR. The ArchR Project contained the filtered cells that had a TSS enrichment below 3 and <1000 fragments. We utilized a layered dimensionality reduction approach using Latent Semantic Indexing (LSI) and Singular Value Decomposition (SVD) applied on Genome-wide tile matrix on the single cell ATAC-seq data. Uniform Manifold approximation and projection (UMAP) was performed to visualize data in 2D space. Louvain Clustering methods implemented in R package Seurat^119^ were used for clustering of the single-cell accessibility profiles. g:Pprofiler GO term analysis of "linked genes" from Day 20 control, P100 and P200 for K-mean clusters 1-5, was performed with g:Profiler R package^137^ displaying results with correlation greater than 0.45_significant. GREAT^135^ pathway enrichment tool was used for analysis of mapped peak to gene links (P2GLs) in k-mean clusters.

### Quantification and statistical analysis

Statistical analyses were performed in R version 4.1.2^117^ applying SARTools v.1.6.8,^152^ DESeq2 v.1.22.1,^128^ Limma^131^, ArchR,^62^ Seurat,^118^^,^^119^ Propeller^53^ and ggpubr package v.0.4.0.^151^ Details are described in the relevant methods sections above.

## Data Availability

• The single-cell RNA-seq/ATAC-seq, RNA-seq and DNA-methylation data reported in this study cannot be deposited in a public repository because the data could be potentially traced back to a single embryo and the donor. To request access, contact the lead author and the Stockholm Medical Biobank. It may be required to establish a Personal Data processing (PDP) Agreement and/or Data Transfer Agreement (DTA) according to General Data Protection Regulation (GDPR). Processed datasets have been deposited at NCBI’s GEO. Accession numbers are listed in the key resources table. Single-cell data are shared for visualization in two open access webtools at https://cancell.medisin.uio.no: (**hescneuroparacet**) https://cancell.medisin.uio.no/scrna/hescneuroparacet/ and (**hescneurodiffparacet**) https://cancell.medisin.uio.no/scatac/hescneurodiffparacet/. • All original code can be found athttps://github.com/EskelandLab/Neuralparacet. DOIs are listed in the key resources table. • Any additional information required to reanalyze the data reported in this paper is available from the lead contact upon request.

## Supplemental information

Document S1. Figures S1–S7

Table S1. Summary of bulk RNA and DNAm analysis, related to Figures 2, 5, 6, S2, and S7 (A) Summary of DEGs and DMCs at all paracetamol comparisons. (B) Differentially expressed genes in cells exposed to 100 μM paracetamol compared to control cells at Day 7. (C) Differentially expressed genes in cells exposed to 200 μM paracetamol compared to control cells at Day 7. (D) Differentially expressed genes in cells exposed to 100 μM paracetamol compared to 200 μM paracetamol cells at Day 7. (E) Differentially expressed genes in cells exposed to 100 μM paracetamol compared to control cells at Day 20. (F) Differentially expressed genes in cells exposed to 200 μM paracetamol compared to control cells at Day 20. (G) Differentially expressed genes in cells exposed to 100 μM paracetamol compared to 200 μM paracetamol cells at Day 20. (H) Differentially expressed genes in cells exposed to 100 μM paracetamol compared to control cells over time (Day 7 - Day 20). (I) Differentially expressed genes in cells exposed to 200 μM paracetamol compared to control cells over time (Day 7 - Day 20). (J) g:Profiler BPs on differentially expressed genes in cells exposed to 100 μM paracetamol compared to control cells over time (Day 7 - Day 20). (K) g:Profiler BPs on differentially expressed genes in cells exposed to 200 μM paracetamol compared to control cells over time (Day 7 - Day 20). (L) Differentially methylated CpGs in cells exposed to 100 μM paracetamol compared to control cells at Day 7. (M) Differentially methylated CpGs in cells exposed to 200 μM paracetamol compared to control cells at Day 7. (N) Differentially methylated CpGs in cells exposed to 100 μM paracetamol compared to control cells at Day 20. (O) Differentially methylated CpGs in cells exposed to 200 μM paracetamol compared to control cells at Day 20. (P) Differentially methylated CpGs in cells exposed to 100 μM paracetamol compared to 200 μM paracetamol cells at Day 20. (Q) Differentially methylated CpGs in cells exposed to 200 μM paracetamol compared to control cells over time (Day 7 - Day 20).

Table S2. scRNA-seq differential expression analysis between paracetamol and control cells, related to Figures 3, S3, S4, and S5 (A) scRNA-seq cluster summary. (B) scRNA differential expression at Day 7 between Control and 100 μM paracetamol. (C) scRNA differential expression at Day 7 between Control and 200 μM paracetamol. (D) scRNA differential expression at Day 7 between 100 μM and 200 μM paracetamol. (E) scRNA differential expression at Day 20 between Control and 100 μM paracetamol. (F) scRNA differential expression at Day 20 between Control and 200 μM paracetamol. (G) scRNA differential expression at Day 20 between Control and 200 μM paracetamol. (H) Overlapping bulk and single cell DEGs. (I) Propeller analysis of cell cluster proportions upon Paracetamol exposure (P100 + P200) compared to control at Day 7. (J) Propeller analysis of cell cluster proportions upon paracetamol exposure (P100 + P200) compared to control at Day 20.

Table S3. Inferred peak to gene links and GO-term enrichment Day 20, related to Figures 4 and S6 (A) Inferred peak to gene links at Day 20 for Control cells. (B) Inferred peak to gene links at Day 20 for P100 exposed cells. (C) Inferred peak to gene links at Day 20 for P200 exposed cells. (D) GOProfiler GO terms for Kmeans 1–5 at Day 20 Control cells, correlation greater than 0.45_significant. (E) GOProfiler GO terms for Kmeans 1–5 at Day 20 P100 exposed cells, correlation greater than 0.45_significant. (F) GOProfiler GO terms for Kmeans 1–5 at Day 20 P200 exposed cells, correlation greater than 0.45_significant. (G) Gene ontology annotation for peak2genelink k-mean cluster 1 for Day 20 Control cells using GREAT Tool. (H) Gene ontology annotation for peak2genelink k-mean cluster 2 for Day 20 Control cells using GREAT Tool. (I) Gene ontology annotation for peak2genelink k-mean cluster 3 for Day 20 Control cells using GREAT Tool. (J) Gene ontology annotation for peak2genelink k-mean cluster 4 for Day 20 Control cells using GREAT Tool. (K) Gene ontology annotation for peak2genelink k-mean cluster 5 for Day 20 Control cells using GREAT Tool. (L) Gene ontology annotation for peak2genelink K-mean cluster 1 for Day 20 P100 cells using GREAT Tool. (M) Gene ontology annotation for peak2genelink K-mean cluster 2 for Day 20 P100 cells using GREAT Tool. (N) Gene ontology annotation for peak2genelink k-mean cluster 3 for Day 20 P100 cells using GREAT Tool. (O) Gene ontology annotation for peak2genelink k-mean cluster 4 for Day 20 P100 cells using GREAT Tool. (P) Gene ontology annotation for peak2genelink k-mean cluster 5 for Day 20 P100 cells using GREAT Tool. (Q) Gene ontology annotation for peak2genelink k-mean cluster 1 for Day 20 P200 cells using GREAT Tool. (R) Gene ontology annotation for peak2genelink k-mean cluster 2 for Day 20 P200 cells using GREAT Tool. (S) Gene ontology annotation for peak2genelink k-mean cluster 3 for Day 20 P200 cells using GREAT Tool. (T) Gene ontology annotation for peak2genelink k-mean cluster 4 for Day 20 P200 cells using GREAT Tool. (U) Gene ontology annotation for peak2genelink k-mean cluster 5 for Day 20 P200 cells using GREAT Tool. (V) Comparison of linked genes (peak2genelinks) of Day 20 Control with P100 and P200 exposure.

Table S4. Inferred peak to gene links TF motif enrichment, related to Figures 4 and S6 (A) TF motif enrichment in the five interaction clusters (k-mean clusters 1–5) corresponding to the Day 20 Control cells. (B) TF motif enrichment in the five interaction clusters (k-mean clusters 1–5) corresponding to the Day 20 P100 cells. (C) TF motif enrichment in the five interaction clusters (k-mean clusters 1–5) corresponding to the Day 20 P200 cells. (D) TF motif enrichment in the five interaction clusters (k-mean clusters 1–5) specific to or intersecting between the Day 20 control cells, Day P100 and P200 cells.
