# Supplementary material for: Multi-omics approach reveals dysregulated genes during hESCs neuronal differentiation exposure to paracetamol
Source: iScience. 2023 Aug 28;26(10):107755. doi: 10.1016/j.isci.2023.107755 (PMC10507163; doi:10.1016/j.isci.2023.107755)
Supplement: Document S1. Figures S1–S7 [file mmc1.pdf]

iScience, Volume *26*

Supplemental information

Multi-omics approach reveals dysregulated genes during hESCs neuronal differentiation exposure to paracetamol

Mari Spildrejorde, Athina Samara, Ankush Sharma, Magnus Leithaug, Martin Falck, Stefania Modafferi, Arvind Y.M. Sundaram, Ganesh Acharya, Hedvig Nordeng, Ragnhild Eskeland, Kristina Gervin, and Robert Lyle

Supplemental Figures

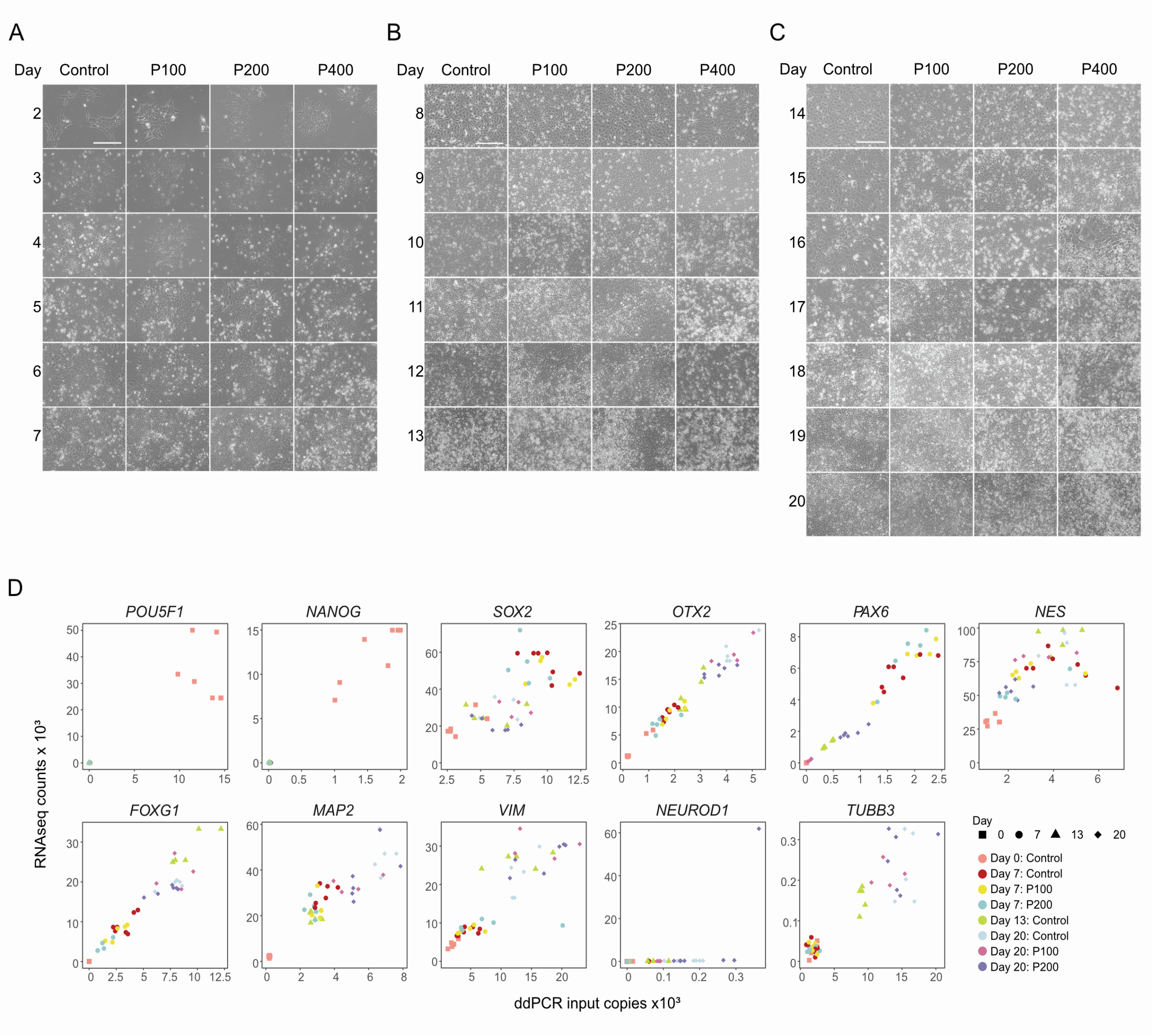

Figure S1. Differentiation timeline for control cells and cells treated with 100, 200 or 400 µM paracetamol. Related to Figures 1 and 2. Brightfield images of control cells, P100, P200 or 400 µM paracetamol (P400) differentiation A) Day 2-7, B) Day 8-13 and C) Day 14-20. Images were taken with an EVOS FL microscope at 20X magnification. Scale bar corresponds to 100 µm. D) ddPCR input copies versus bulk mRNA expression of selected marker genes from Days 0, 7, 13 and 20 (square, circle, triangle and diamond, respectively) in control cells and cells exposed to 100 or 200 µM paracetamol (represented by different colours). One dot represents one replicate.

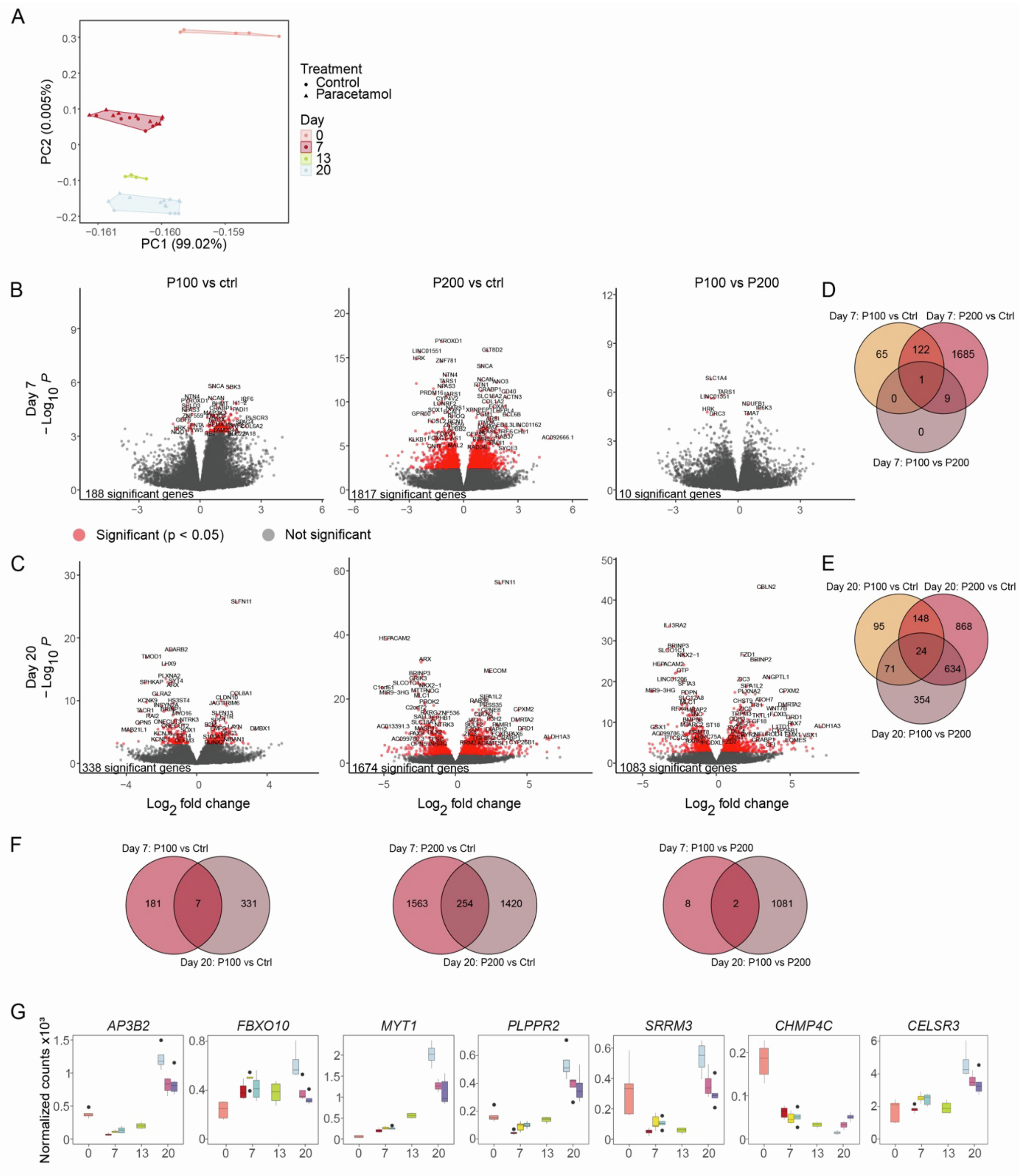

Figure S2. Global gene expression analysis of differentiating neuronal cells exposed to paracetamol. Related to Figure 2. A) Principal component analysis of replicates coloured by day and exposure group. B-C) Volcano plots showing differentially expressed genes between cells treated with 100 µM paracetamol compared to control (left), 200 µM paracetamol compared to control (middle) and 100 µM paracetamol compared to 200 µM paracetamol (right) at B) Day 7 and C) Day 20. Venn diagrams showing number of overlapping DMCs between D) Day 7 comparisons, E) Day 20 comparisons and F) Day 7 and Day 20 comparisons. Genes with FDR < 0.05 are considered significant. G) Gene expression levels of selected DEGs, which overlapped between time-response (TR) P100 and TR P200.

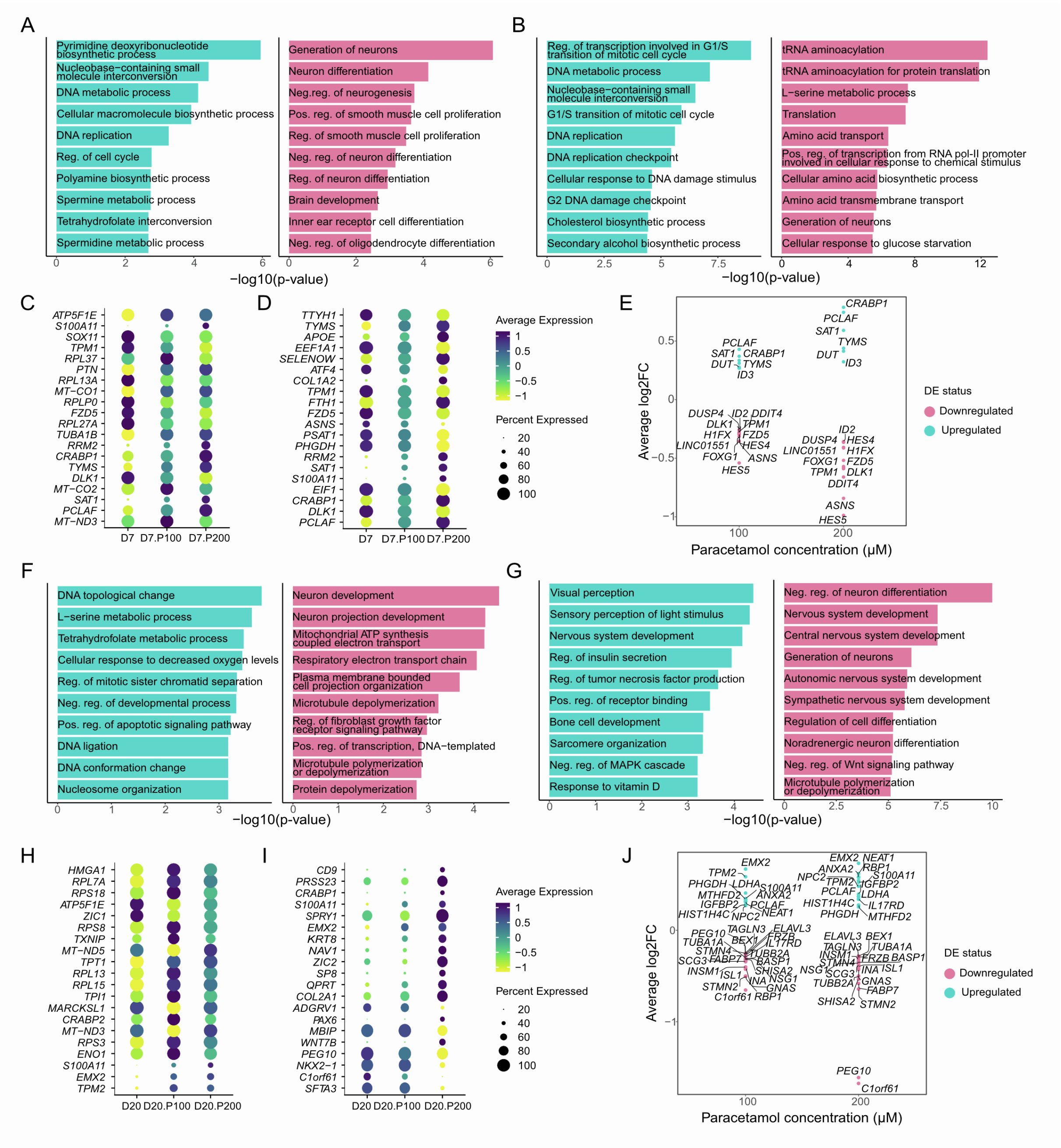

Figure S3. Differential gene expression analysis in single cells showed downregulation of genes involved in neuronal differentiation after paracetamol exposure. Related to Figure 3. A-B) Top 10 upregulated (green) and downregulated (pink) BPs among DEGs at Day 7 between A) P100 or B) P200 and control cells. Bubble plot showing gene expression for the top 20 DEGs at Day 7 between C) P100 or D) P200 and control cells. E) Gene expression of top overlapping genes between P100 and P200 cells compared to control cells at Day 7. F-G) Top 10 upregulated (green) and downregulated (pink) BPs of DEGs at Day 20 between F) P100 or G) P200 and control cells. Bubble plot showing gene expression for the top 20 DEGs at Day 20 between H) P100 or I) P200 and control cells. J) Gene expression of top overlapping genes between P100 and P200 cells compared to control cells at Day 20.

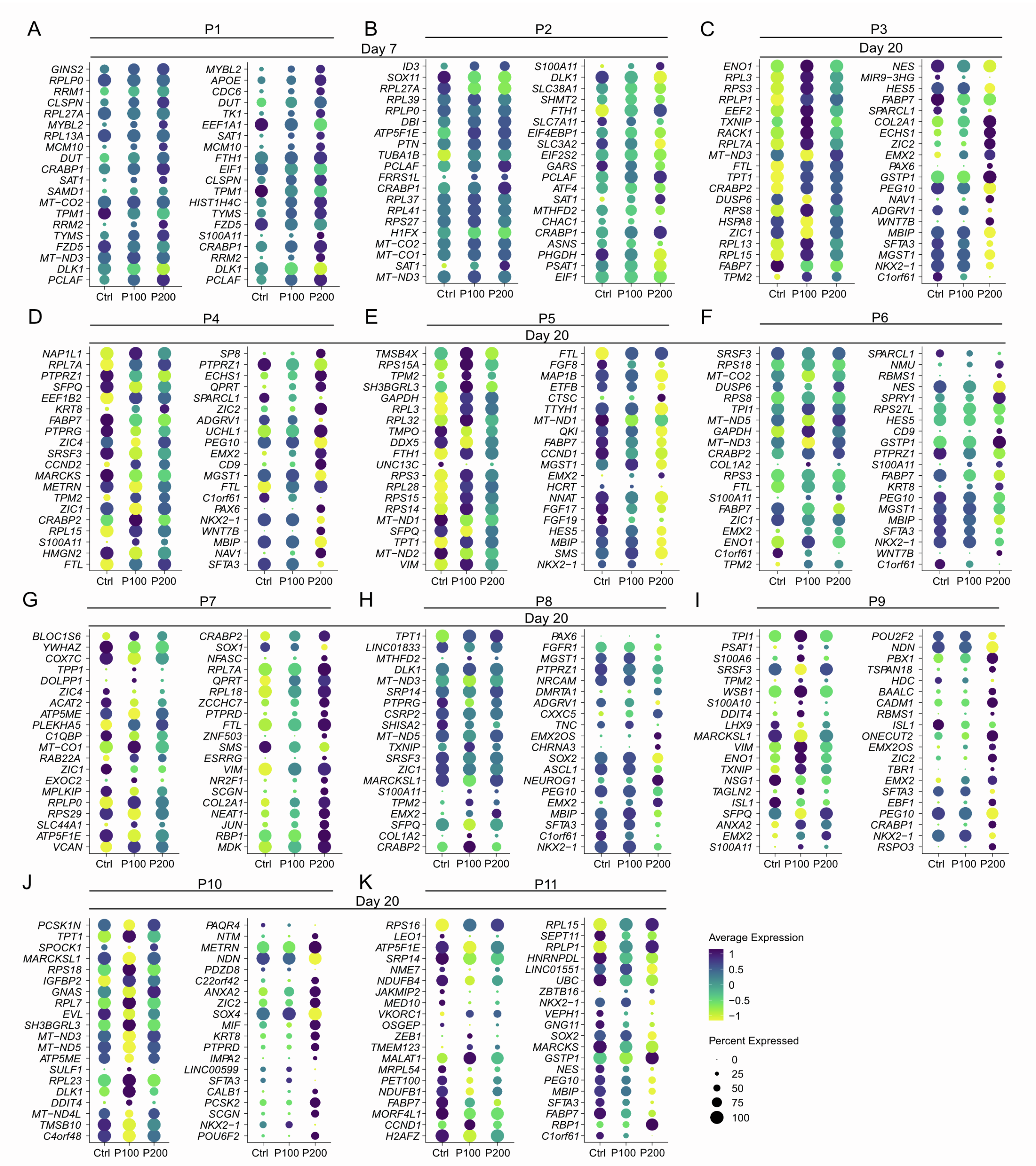

Figure S4. Bubble plots of single-cell differential expression analysis per cluster. Related to Figure 3. Gene expression of the top 20 differentially expressed genes between P100 cells and control (left) and P200 cells and control (right) in cluster A) P1, B) P2, C) P3, D) P4, E) P5, F) P6, G) P7, H) P8, I) P9, J) P10 and K) P11. Plots compare differentially expressed genes either between Ctrl and P100 or between Ctrl and or P200 per cluster; however, the levels of both P100 and P200 are included for reference.

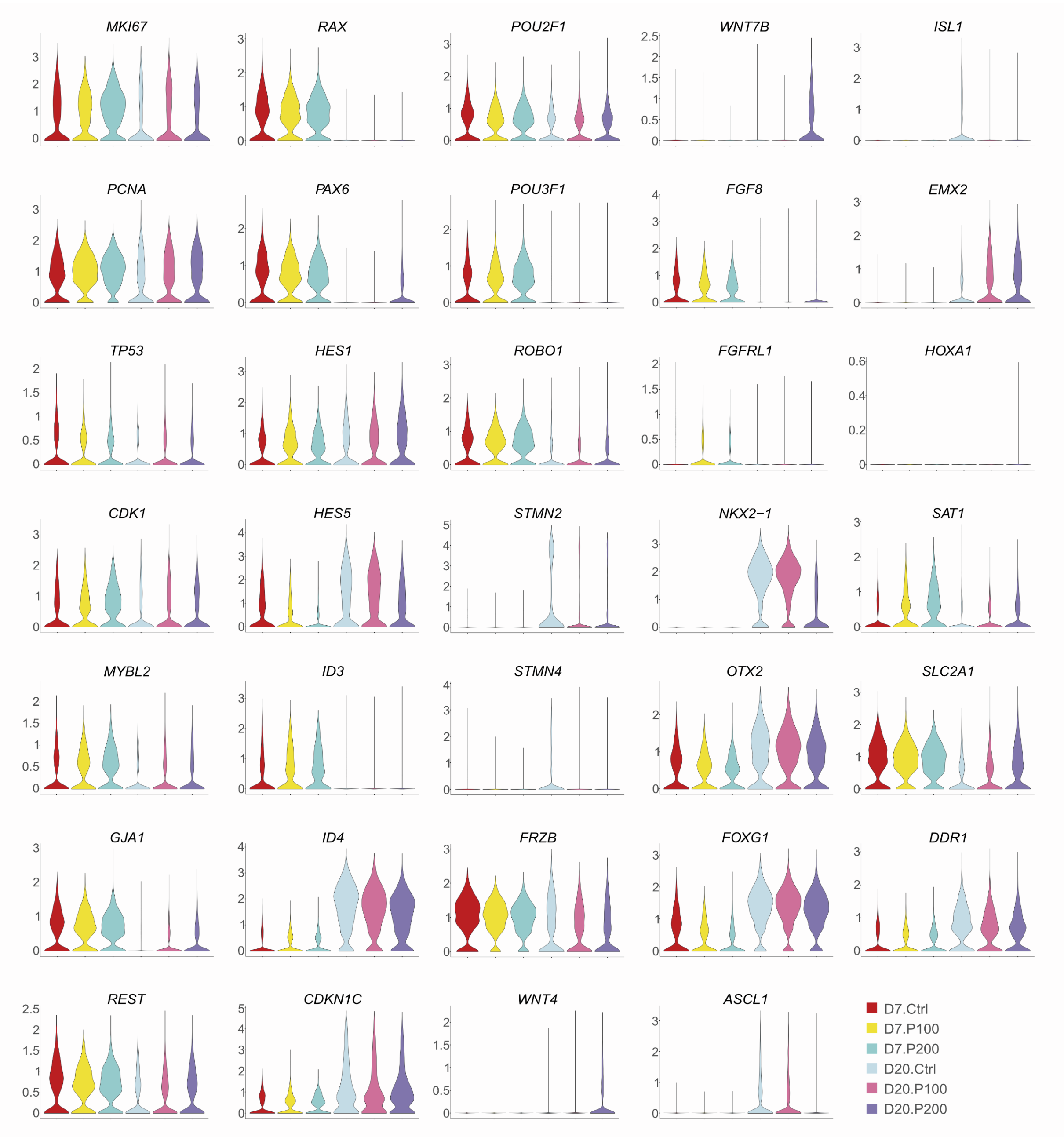

Figure S5. Gene expression changes in paracetamol-exposed compared to control cells in scRNA data. Related to Figure 3. Gene expression of selected differentially expressed genes *MKI167*, *PCNA*, *TP53*, *CDK1*, *MYBL2*, *GJA1*, *REST*, *RAX*, *PAX6*, *HES1*, *HES5*, *ID3*, *ID4*, *CDKN1C*, *POU2F1*, *POU3F1*, *ROBO1*, *STMN2*, *STMN4*, *FRZB*, *WNT4*, *WNT7B*, *FGF8*, *FGFRL1*, *NKX2-1*, *OTX2*, *FOXG1*, *ASCL1*, *ISL1*, *EMX2*, *HOXA1*, *SAT1*, *SLC2A1* and *DDR1*.

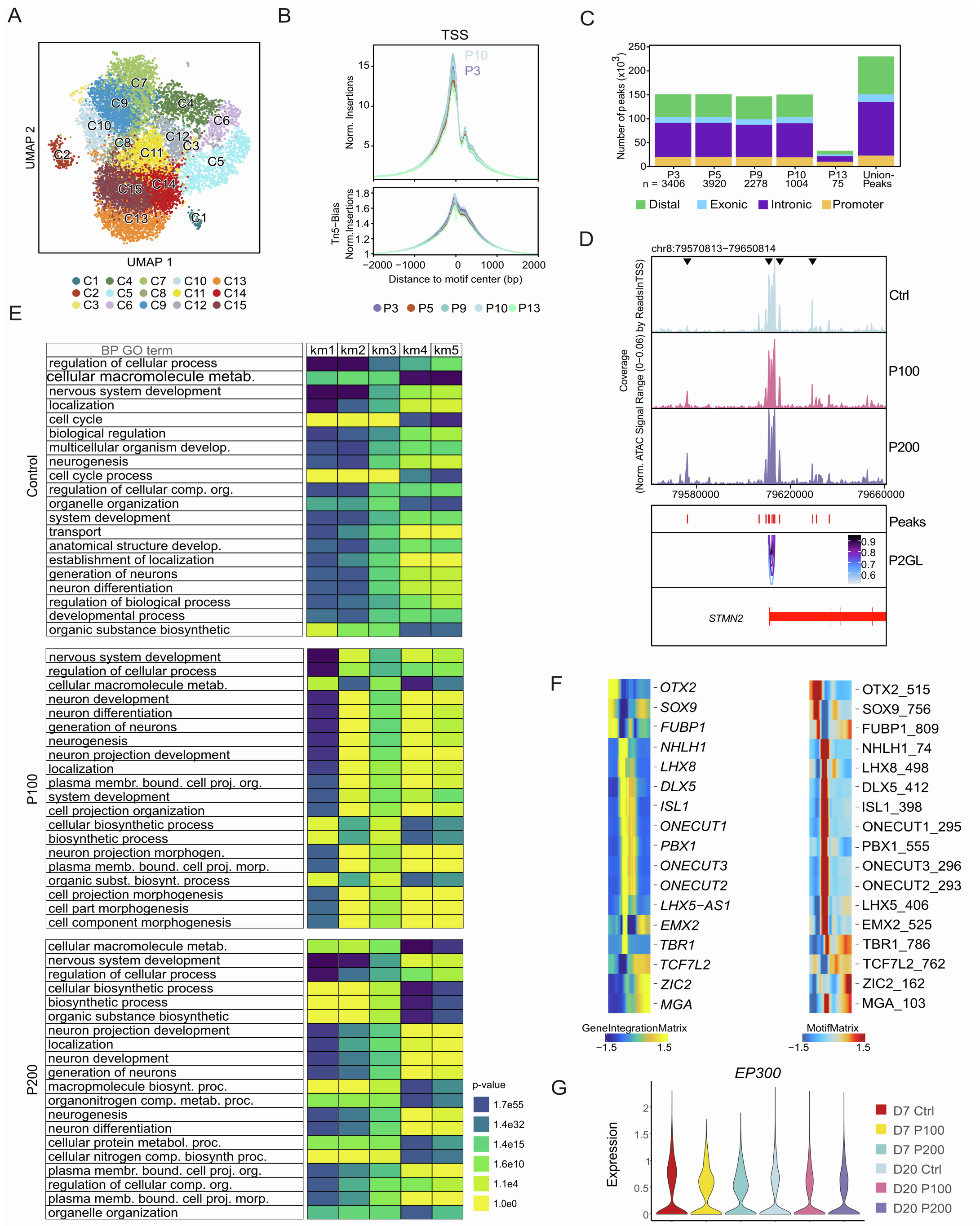

Figure S6. Integrative chromatin accessibility analysis in neuronal differentiation Day 20 upon paracetamol exposure. Related to Figure 4. A) UMAP plot representing clusters at scATAC-seq modality (C1-C15). B) Chromatin opening across all gene TSS in integrated clusters P3, P5, P9, P10 and P13. Tn5 bias normalized insertions are shown below. C) Bar plot of number of peaks in distal genomic regions, exons, introns, and promoters. D) *STMN2* locus browser view (GRCh38.p13) with ATAC-seq signals generated from 5000 cells for control, P100 and P200 cells. ATAC peaks (red) and P2GLs (arcs, blue gradient) are shown. Black arrows indicate some peaks where change in ATAC-seq signals between control, P100 and P200 can be observed. E) Top 20 significant GO terms of controls, P100 and P200 linked genes (active genes linked with putative CREs having correlation value greater than 0.45 and significant FDR<1e-4) for k - mean groups 1-5. F) Side-by-side heatmaps of top transcription factor regulators where gene expression is positively correlated with chromVAR deviation computed on Gene integration (left) and Motif Matrix (right) for all Day 20 cells. G) scRNA gene expression of *EP300* at Day 7 and Day 20 P100, P200 and control cells.

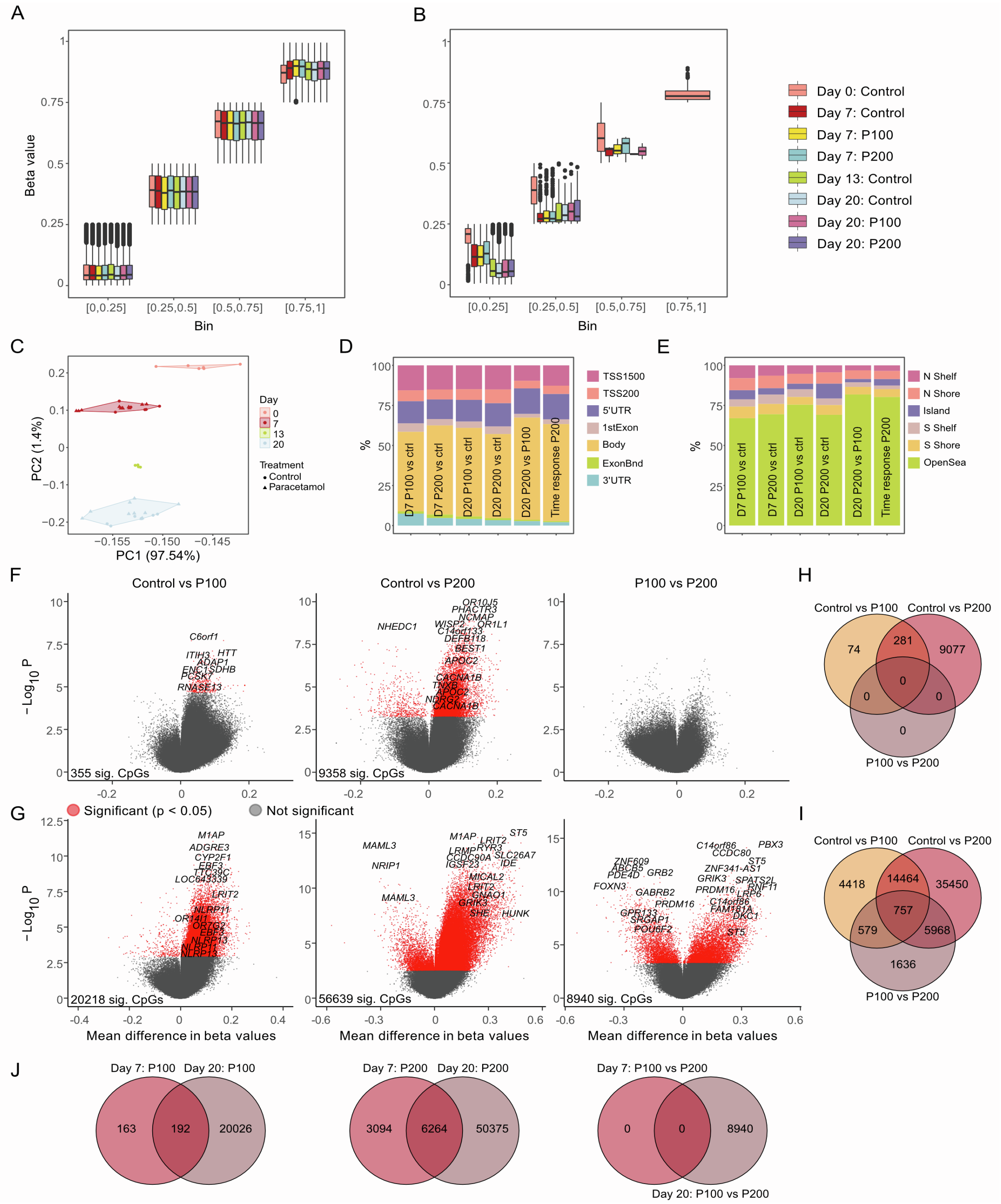

Figure S7. DNA methylation analysis of neuronal differentiation during paracetamol exposure. Related to Figures 5 and 6. A) Average DNAm levels for each sample across all CpGs and non-CpGs (grouped in bins of 0.25) at different days in control cells and cells exposed to different paracetamol doses. B) Average DNAm levels for each sample across all non-CpGs (grouped in bins of 0.25) for all controls and exposed cells. C) Principal component analysis of replicates coloured by day and exposure group. Distribution of significant CpGs in relation to D) annotated genes and E) CpG islands for the different comparisons. F-G) Volcano plots showing DMCs between P100 cells compared to control (left), P200 compared to control (middle) and P100 compared to P200 (right) at F) Day 7 and G) Day 20. CpGs with FDR > 0.05 are considered significant. Venn diagrams showing number of overlapping DMCs between H) Day 7 comparisons, I) Day 20 comparisons and J) Day 7 and Day 20 comparisons. CpGs with FDR < 0.05 are considered significant.
